# Supplementary material for: Light-induced MOF synthesis enabling composite photothermal materials
Source: Nat Commun. 2024 Feb 7;15:1154. doi: 10.1038/s41467-024-45333-9 (PMC10850081; doi:10.1038/s41467-024-45333-9)
Supplement: Supplementary file 1 — Supplementary Information [file 41467_2024_45333_MOESM1_ESM.pdf]

Supplementary Information

Light-induced MOF synthesis enabling composite photothermal materials

Ofir Shelonchik[1], Nir Lemcoff[1], Ran Shimoni[1], Aritra Biswas[1], Elad Yehezkel[1], Doron Yesodi[1], Idan Hod[1,2,3] and Yossi Weizmann[1,2,3]*

[1]Department of Chemistry, Ben-Gurion University of the Negev, Beer-Sheva 84105, Israel
[2]Ilse Katz Institute for Nanotechnology Science, Ben-Gurion University of the Negev, Beer-Sheva 84105, Israel
[3]Goldman Sonnenfeldt School of Sustainability and climate Change, Ben-Gurion University of the Negev, Beer-Sheva 84105, Israel
*Correspondence to: yweizmann@bgu.ac.il (Y.W.)

Table of Contents

1. Supplementary Note 1: List of Materials 4
2. Supplementary Note 2: Instruments 4
3. Supplementary Note 3: AuBPs Characterization 5
3.1 Supplementary Figure 1: UV-Vis spectrum. 5
3.2 Supplementary Figure 2-5: TEM images 6
3.3 Supplementary Figure 6-7: Uncoated AuBPs 10
4. Supplementary Note 4: Photo-induced MOF synthesis 11
4.1 Supplementary Figure 8: System setup 11
4.2 Supplementary Figure 9: Photothermal synthesis of UIO-66 using 8W 850nm LED 12
5. Supplementary Table 1: T_{max} of PPR of UIO-66 13
6. Supplementary Note 5: Scaled-up photothermal synthesis of UIO-66 13
6.1 Supplementary Table 2 13
6.2 Supplementary Figures 10-12 13
7. Supplementary Note 6: Photothermal synthesis of UIO-66 at different temperature 15
7.1 Supplementary Figures 13-15 15
7.2 Supplementary Table 3 16
7.3 Supplementary Figures 16-27: SEM images of AuBP@UIO-66 17
7.4 Supplementary Table 4: ICP-OES of AuBPs@UIO-66 23
7.5 Supplementary Figures 28-30: TEM images of AuBP@UIO-66 23
7.6 Supplementary Table 5: Surface areas of AuBP@UIO-66 25
7.7 Supplementary Figure 31-33: Size distribution of UIO-66 particles 26
8. Supplementary figure 34: Zeta potential 28
9. Supplementary Note 7: AuBPs@SiO_2 and zirconium interaction 28
9.1 Supplementary figure 35-37: AuBPs with $ZrCl_4$ at different temperatures 28
9.2 Supplementary Figure 38-40: STEM images and EDS analysis 30
9.3 Supplementary Figure 41: X-ray photoelectron spectroscopy 32
9.4 Supplementary Figure 42: PXRD pattern of AuBPs covered with zirconium oxide. 33
9.5 Supplementary Figure 43-44: TEM images of AuBPs@Zr@UIO-66 34
10. Supplementary Figure 45-46: UIO-66 synthesis using methylene blue 35
11. Supplementary Figure 47-48: Conventional synthesis of UIO-66 36
12. Supplementary Note 8: $AuBP_{850}$ recycling 37
12.1 Supplementary Figure 49: Temperature profile 37
12.2 Supplementary Table 6: UIO-66 mass 37
13. Supplementary Note 9: Versatility and scope of MOFs photothermal synthesis 38
13.1 Supplementary Figure 50-54: Photothermal synthesis using Carbon Black (CB) 38

13.2 Supplementary Figure 55-56: Photothermal synthesis using Activated charcoal (AC) 41
13.3 Supplementary Figure 57-58: Photothermal synthesis using graphene oxide............................42
13.4 Supplementary Figure 59-61: Photothermal synthesis using $AuBPs_{660}$.......................................43
13.5 Supplementary Figures 62-66: Photothermal synthesis using AuNS 45
13.6 Supplementary Figures 67-71: Photothermal synthesis using AuNR 47
14. Supplementary Note 10: AuBP embedded UIO-66 50
14.1 Supplementary Figure 72: Heating ability of UIO-66 without AuBPs 50
14.2 Supplementary Table 7: Heating-Cooling cycles properties...50
14.3 Supplementary Figure 73-74 Heating-Cooling cycles 51
14.4 Supplementary Figures 75-76: Water desorption 52
15. Supplementary Note 11: Photothermal activation 53
15.1 Supplementary Figure 64: Photothermal activation set-up 53
16. Supplementary Note 12: UIO-66@UIO-66 54
16.1 Supplementary Figure 78: Temperature profile UIO-66@UIO-66 synthesis cycles. 54
16.2 Supplementary Table 8: UIO-66@UIO-66 synthesized masses 54
17. Supplementary references 55

1. Supplementary Note 1: List of Materials

All materials were purchased from Sigma-Aldrich unless noted otherwise. Ultrapure water (type 1, 18.2 MΩ) from Millipore® Direct-Q® 3 with UV was used. Cetyltrimethylammonium chloride (CTAC) sodium borohydride ReagentPlus 99 %, sodium citrate tribasic BioUltra ≥99.5 %, gold chloride trihydrate 99.9 %, cetyltrimethylammonium bromide (CTAB) ≥99 %, ascorbic acid BioXtra ≥99.0 %, hydrochloric acid 32 %, silver nitrate BioXtra ≥99 %, tetraethyl orthosilicate (TEOS) reagent grade 98 %, ammonium hydroxide 28 % in water 99.9 %, N,N-Dimethylformamide (DMF) 99.8% for spectroscopy Acros Organics, Zirconium(IV) chloride ($ZrCl_4$,≥99.5%), Terephthalic acid (BDC) 98%, Copper nitrate trihydrate ($Cu(NO_3)_2{\cdot}3H_2O$) 99% for analysis ThermoScientific, BTC, Ethanol (EtOH) 99.9% tech Romical, Iron(III) chloride hexahydrate ($FeCl_3{\cdot}6H_2O$) ACS reagent 97%, Fumaric acid 99+% Acros Organics, Zinc nitrate hexahydrate ($Zn(NO_3)_2{\cdot}6H_2O$) 98% Thermo scientific, Carbon black, acetylene, 50% compressed, 99.9%, Thermo Scientific – Alfa Aesar, Graphene oxide sheets. Methylene blue hydrate, 97.0%.

2. Supplementary Note 2: Instruments

Ultra-violet visible (UV-Vis) light spectrophotometer

Thermos-Scientific Evolution 220 UV-Visible spectrophotometer was used to determine AuBPs absorbance solution's localized surface plasmon resonance (LSPR) activation wavelength and optical density (OD).

Transmission electron microscope (TEM)

TEM images were obtained using a Thermo Fisher Scientific (FEI) Talos F200C transmission electron microscope operating at 200 kV. The images were taken with Ceta 16M CMOS camera. Samples were prepared by adding 3 µl of an AuBP in ethanol solution (2 OD) on Electron Microscopy Sciences formvar/carbon 200 Mesh, copper grids and let it evaporate under air.
Elemental mapping was performed using JEM-2100F, a field emission gun TEM, operating in scanning transmission electron microscopy (STEM) mode, to record the energy dispersive X-ray spectroscopy (EDS) data with the Oxford EDS system.

Scanning electron microscope (SEM)

UIO-66 SEM images were taken using a Thermo Fisher Scientific Verios 460L FEI scanning electron microscope. Dry samples were put on carbon tape, then coated with carbon coating using an Emitech k575x. HKUST-1, MIL-88A and MOF-5 SEM images were taken using LVEM5 benchtop electron microscope. Dry samples were put on carbon tape.

X-ray diffraction (XRD)

Panalytical Empyrean II Diffractometer system- equipped with three position sensitive detectors: X'celerator 1D, 1der (0D and 1D applications), and PIXcel3D detector (with pre-mounted diffracted beam monochromator).

Breuner-Emmett-Teller (BET)

BET- NOVA touch- Containing 4 degassing stations and up to 4 analysis stations, the instrument delivers four 5-point BETs in 20 mins, with 2 % reproducibility. Samples were degassed for 17 hours at 120 °C.

Inductively Coupled Plasma Optical Emission Spectrometry (ICP-OES)

The amount of Au in AuBP@UIO-66 samples was determined using a SPECTRO ARCOS ICP-OES. The samples were prepared by dissolving AuBPs from AuBP@UIO-66 in a 10 % nitric acid solution, and then were filtered with a 45 μm filter.

3. Supplementary Note 3: AuBPs Characterization

3.1 Supplementary Figure 1: UV-Vis spectrum.

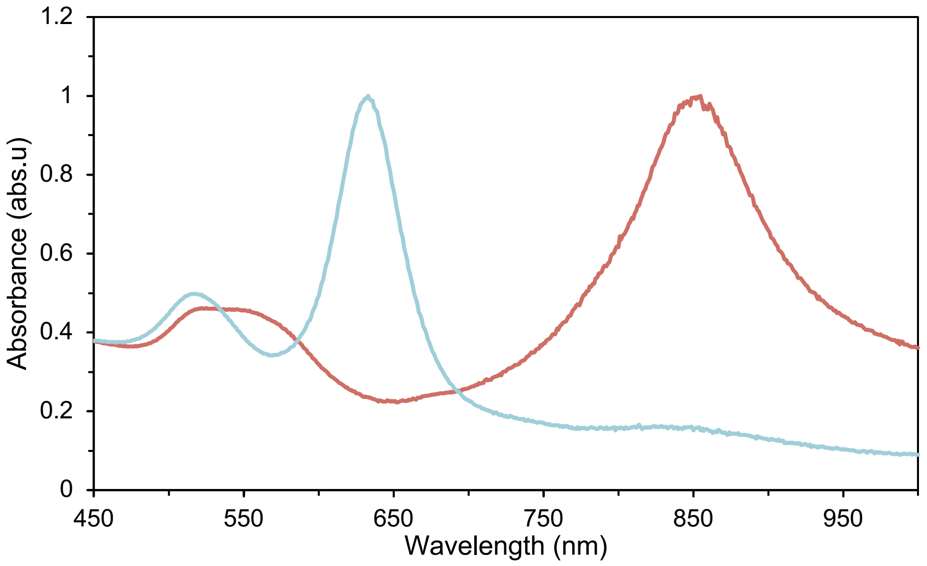

Supplementary figure 1 | Absorption spectrum of **$AuBP_{660}$** (blue line) and **$AuBP_{850}$** (red line).

3.2 Supplementary Figure 2-5: TEM images

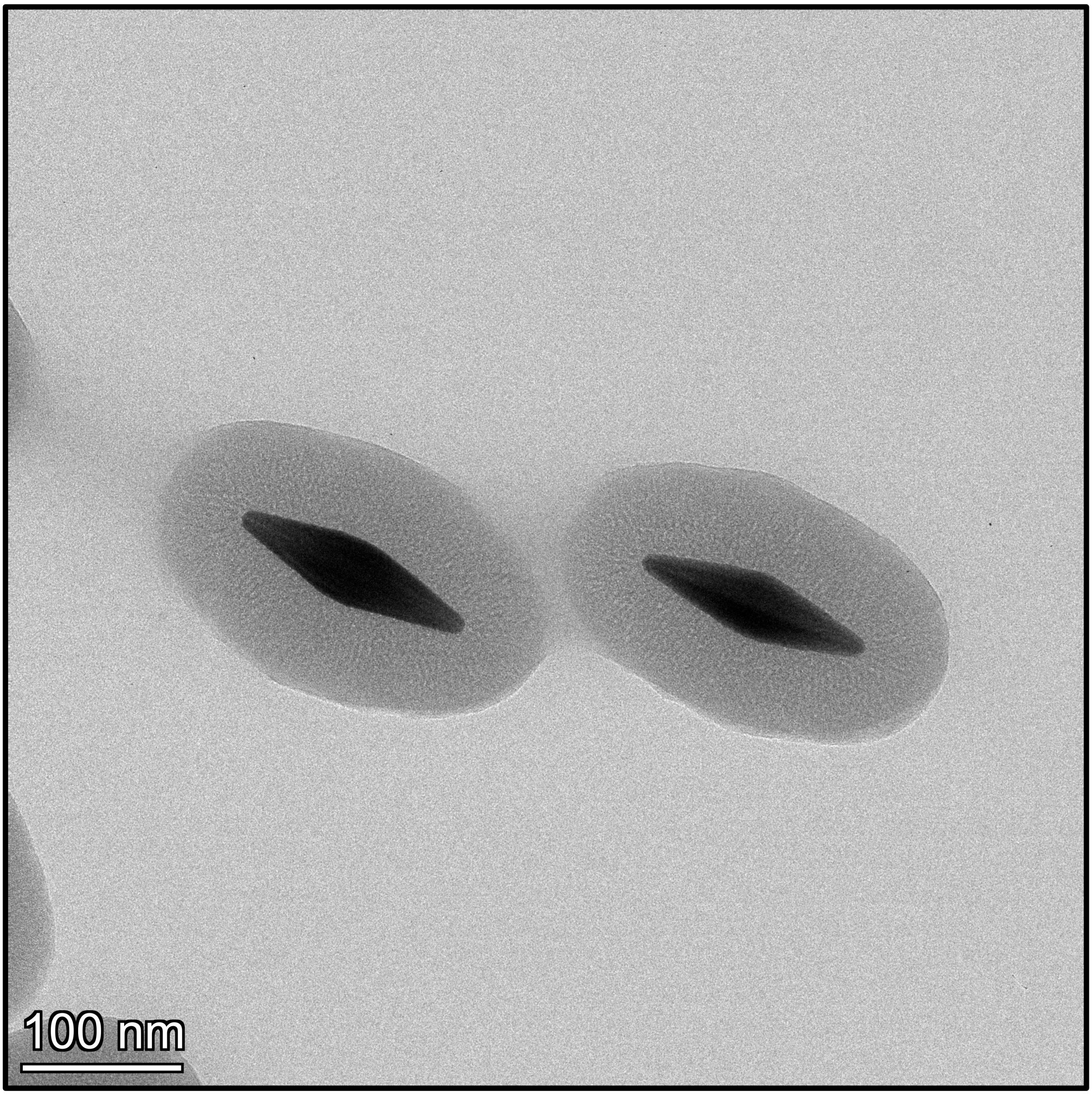

Supplementary figure 2 | TEM image of AuBPs that adsorb light in the IR range (**AuBP$_{850}$**). The darker shape is the gold bipyramid, the rounded grey shape is the silica shell.

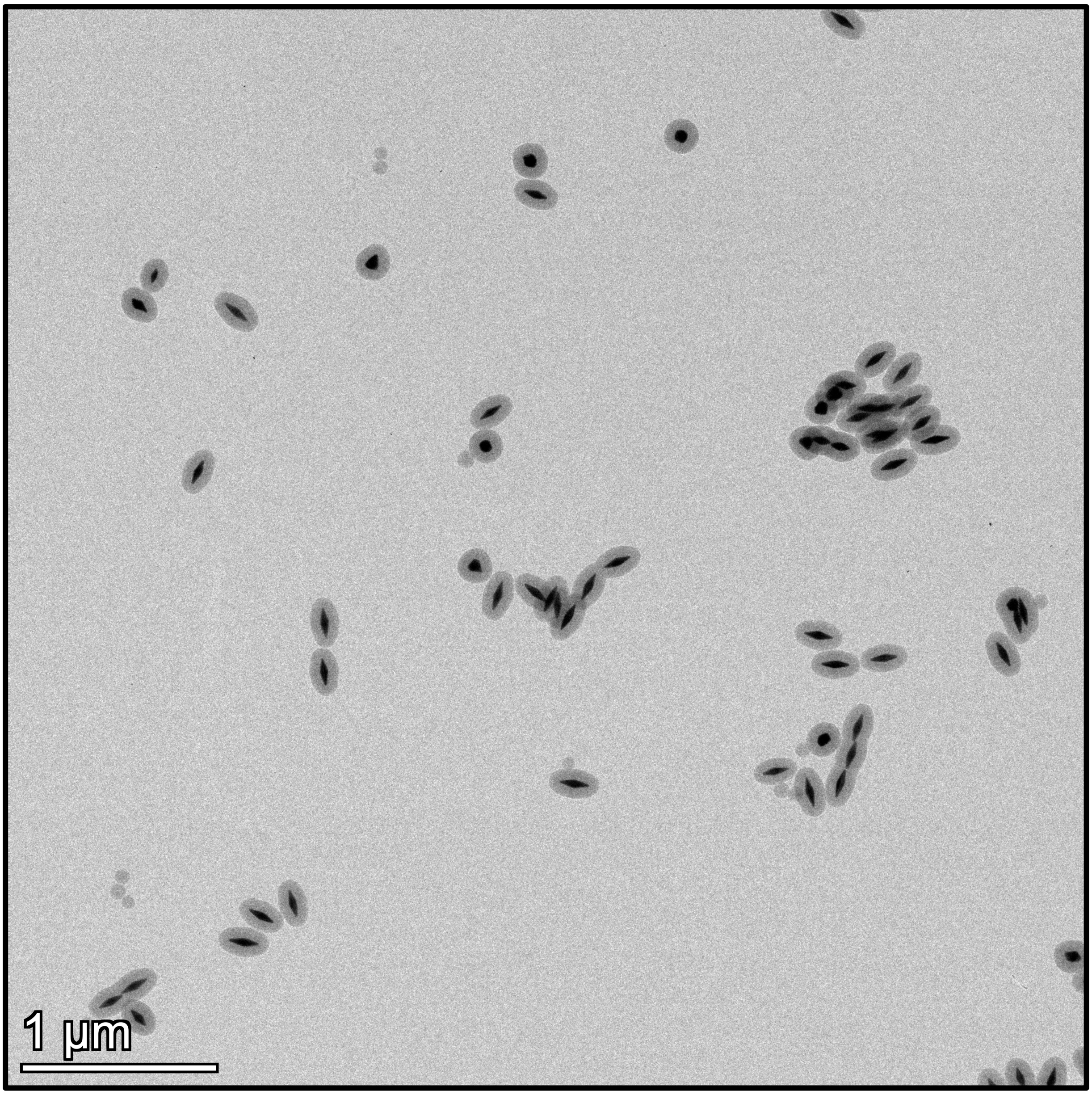

Supplementary figure 3 | Zoomed out TEM image of **AuBP$_{850}$**.

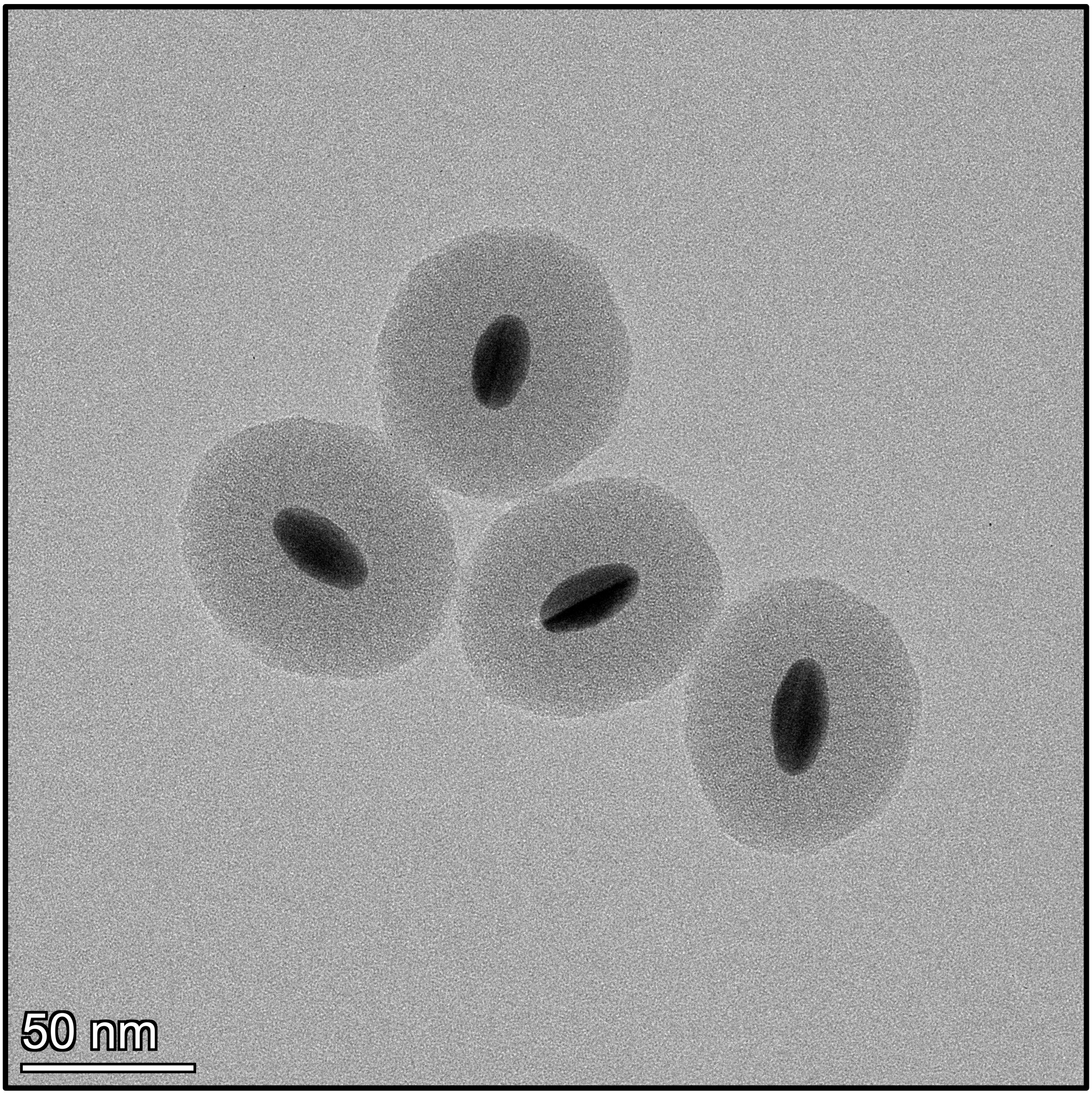

Supplementary figure 4 | TEM image of silica encapsulated AuBPs that adsorb light at 660 nm wavelength (**AuBP$_{660}$**).

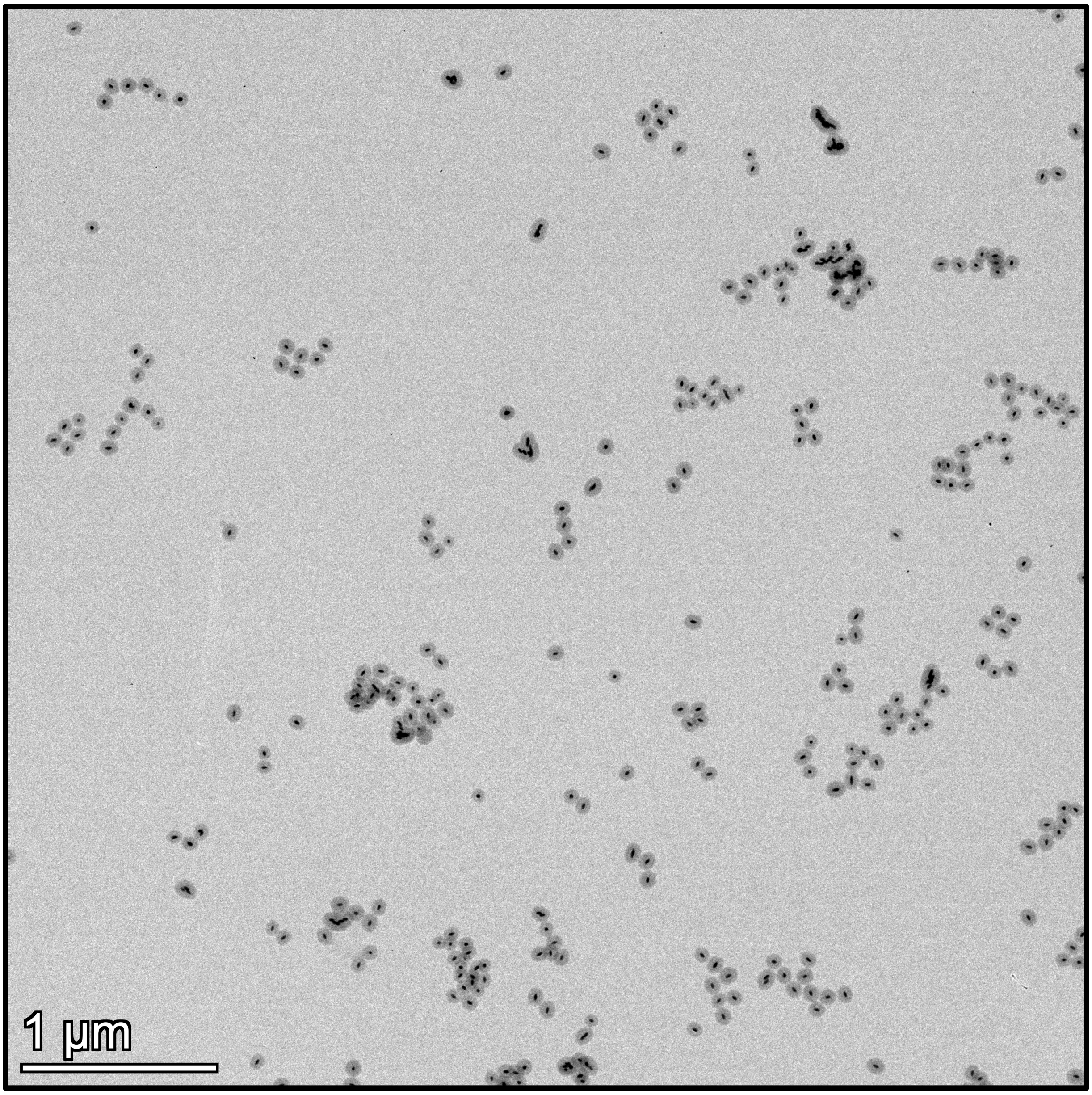

Supplementary figure 5 | Zoomed out TEM image **AuBP$_{660}$**.

3.3 Supplementary Figure 6-7: Uncoated AuBPs

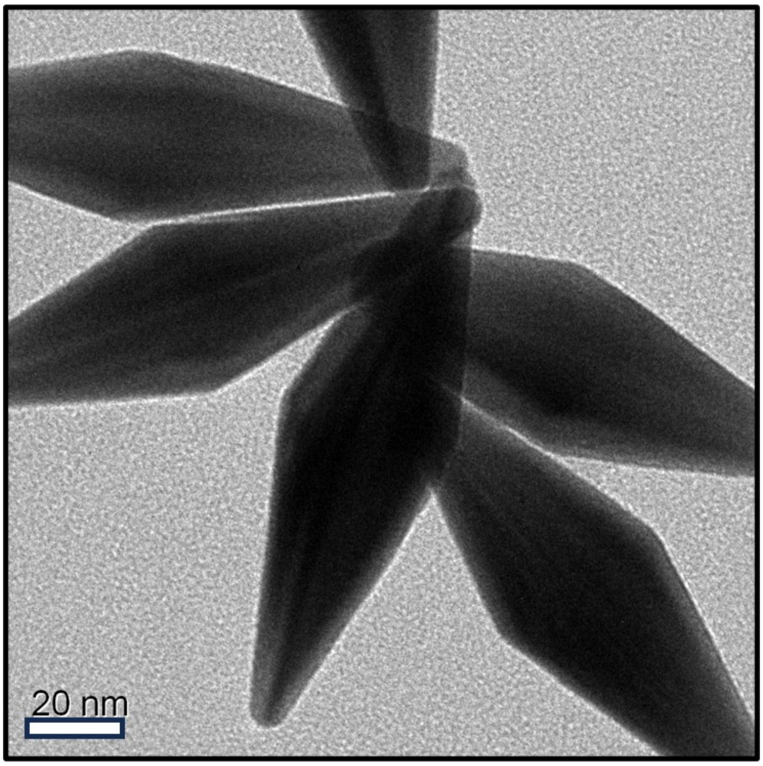

Supplementary figure 6 | TEM image of uncoated AuBPs$_{850}$.

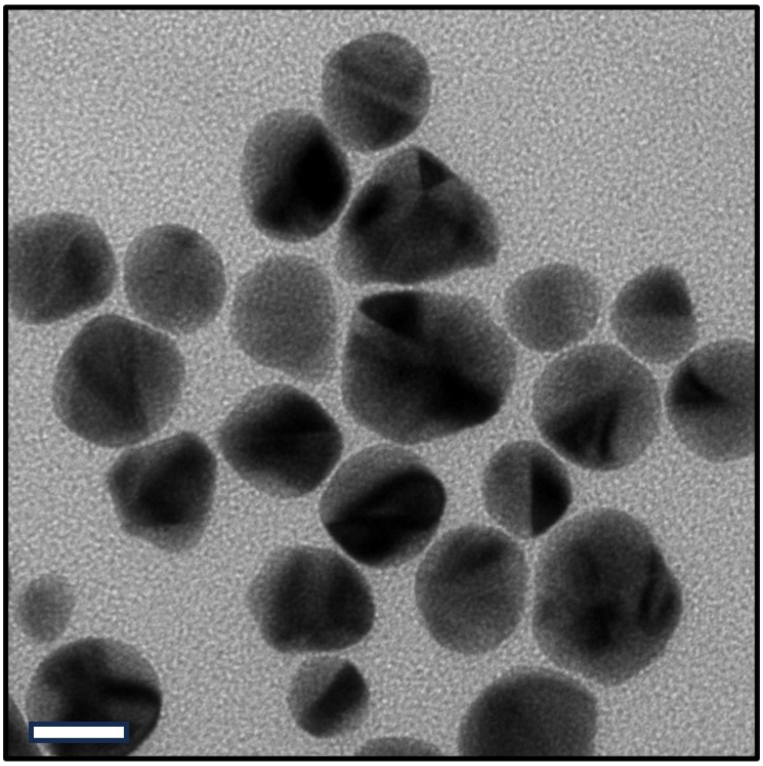

Supplementary figure 7 | TEM image of uncoated AuBPs$_{850}$ after irritated with 100W LED of 850 nm for 1 hour. Scale bar 20 nm.

4. Supplementary Note 4: Photo-induced MOF synthesis

4.1 Supplementary Figure 8: System setup

All the LEDs were purchased from LED ENGIN, 9130B-BK Precision programmable DC power supply was used to connect the LEDs to the computer. The temperature was monitored by MLX90614-DF ROBOT IR Thermometer Sensor, which was connected to the computer with an Arduino Mega. The control on the different parts of the system is gained with a designated LabView program.

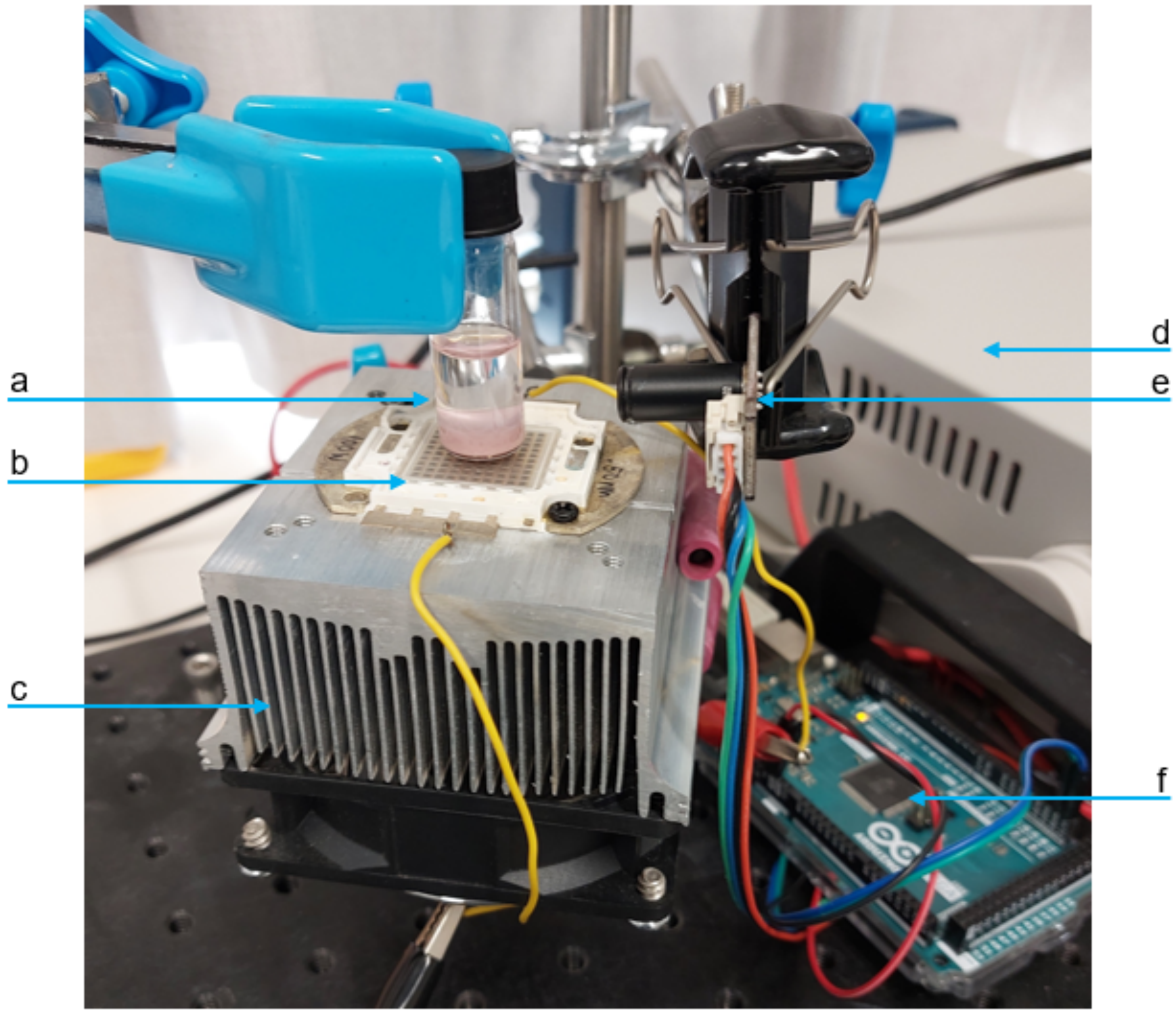

Supplementary figure 8 | a, After a synthesis of $AuBP_{850}$@UIO-66 in 4 mL vial with 2 OD of $AuBPs_{850}$ **b,** LED ENGIN 100 W, 850 nm. **c,** Aluminum heat-sink with a radiator cooling fan. **d,** 9130B-BK. **e,** MLX90614-DF ROBOT IR Thermometer Sensor. **f,** Arduino Mega.

4.2 Supplementary Figure 9: Photothermal synthesis of UIO-66 using 8W 850nm LED

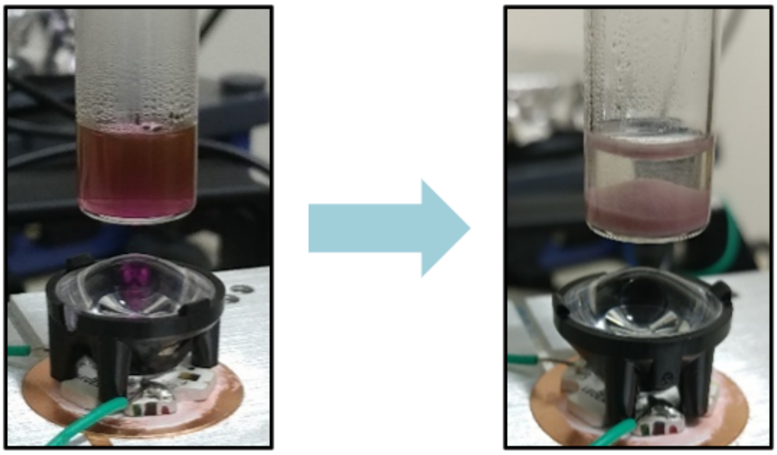

Supplementary figure 9 | Scheme of Photothermal synthesis of UIO-66 with $AuBP_{850}$, using 8W IR LED, the two images display before and after the procedure.

5. Supplementary Table 1: T_{max} of PPR of UIO-66

Supplementary table 1

Entry	AuBP concentration (OD)	T_{max} (°C)	Completion time (min)
1	0.5	99.65	20
2	0.75	109.2	15
3	1	122.8	10
4	2	135.1	5

Photothermal synthesis of UIO-66 with different concentrations of AuBPs

6. Supplementary Note 5: Scaled-up photothermal synthesis of UIO-66

6.1 Supplementary Table 2

Supplementary table 2

Entry	AuBP concentration (OD)	Volume (ml)	Heating method	Temperature (°C)	Time (minutes)
1	0	20	Heating plate	100	60
2	5	20	Heating plate	100	60
3	5	20	Photothermal	100	60

Reaction conditions for the samples described in manuscript figure 1d

6.2 Supplementary Figures 10-12

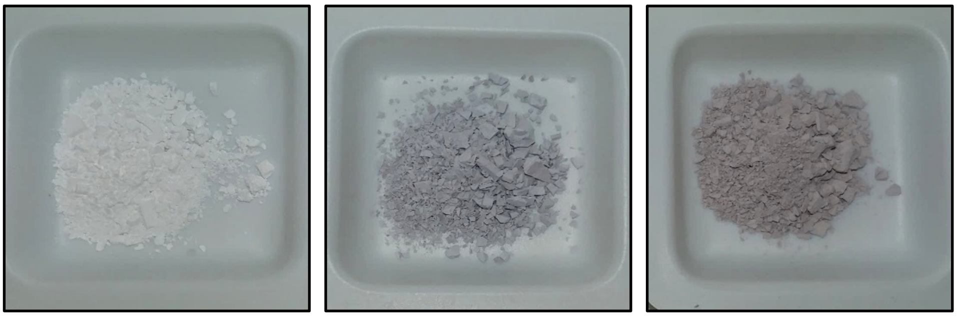

Supplementary figure 10 | Images of the products from different UIO-66 syntheses. From left to right- entry 1, 2, 3, supplementary table 2.

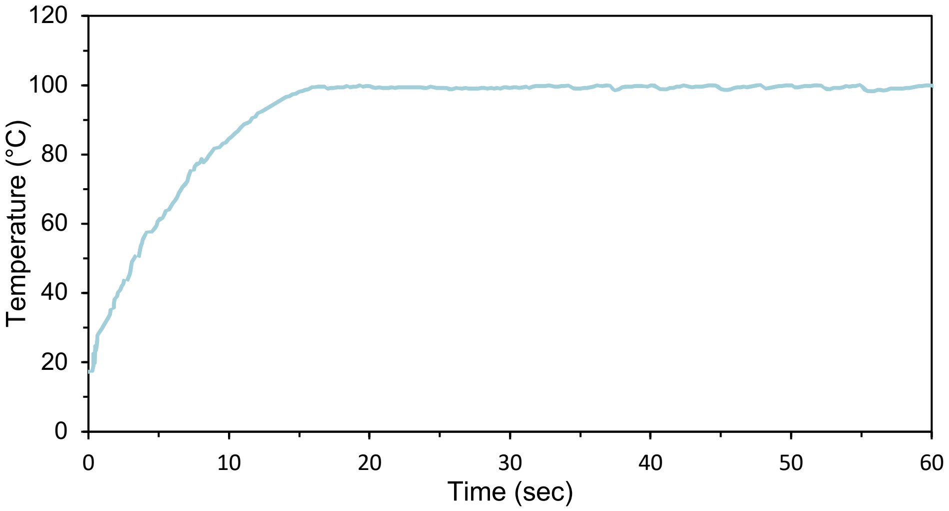

Supplementary figure 11 | Temperature profile of entry 3, supplementary table 2.

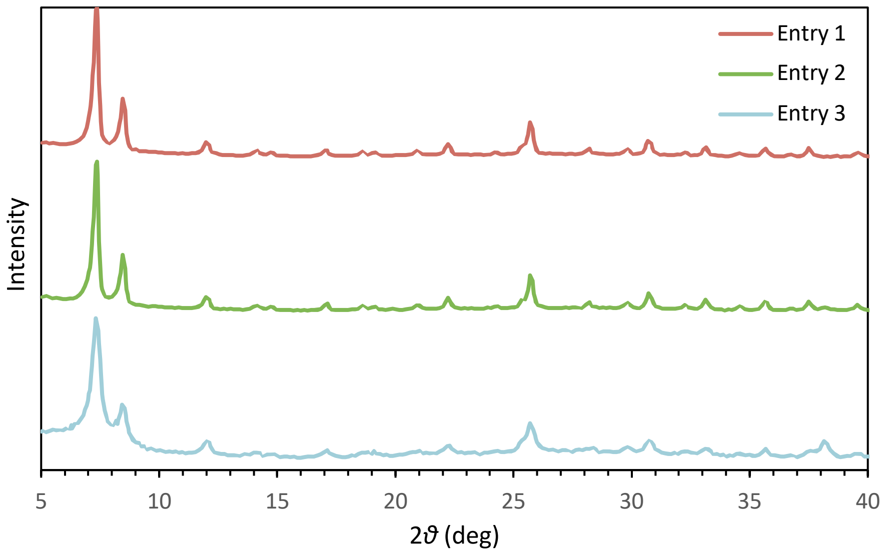

Supplementary figure 12 | PXRD patterns of entry 1,2,3, supplementary table 2.

7. Supplementary Note 6: Photothermal synthesis of UIO-66 at different temperature

7.1 Supplementary Figures 13-15

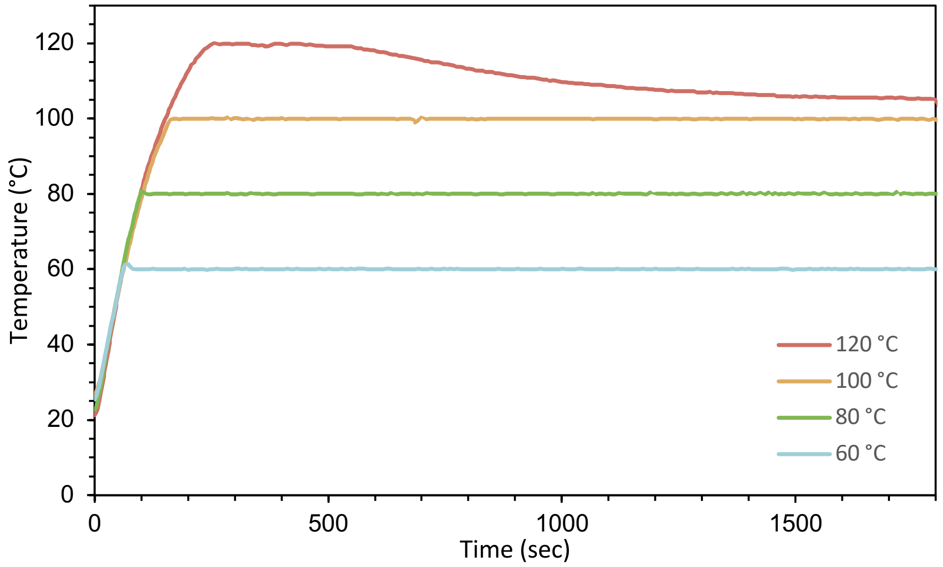

Supplementary figure 13 | Temperature profiles of photothermal syntheses of UIO-66.

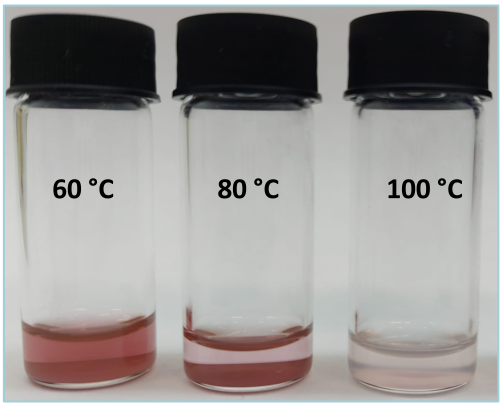

Supplementary figure 14 | Supernatants from PPR of $AuBP_{850}$@UIO-66 synthesized at different temperatures

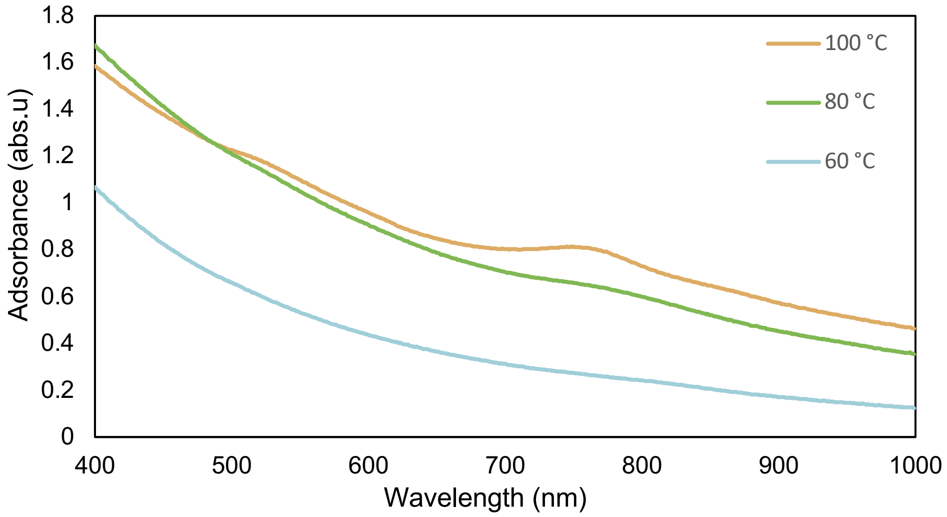

Supplementary figure 15 | UV-vis spectra of $AuBP_{850}$@UIO-66 synthesized at different temperatures

7.2 Supplementary Table 3

Supplementary table 3

Entry	Image	Synthesis temperatures (°C)	Magnification	Mode*
1	a	60	10000	BSE+SE
2		60	10000	SE
3	b	60	20000	BSE+SE
4		60	20000	SE
5	c	80	10000	BSE+SE
6		80	10000	SE
7	d	80	20000	BSE+SE
8		80	20000	SE
9	e	100	10000	BSE+SE
10		100	10000	SE
11	f	100	20000	BSE+SE
12		100	20000	SE

Reaction conditions and image specifications corresponding to the SEM images described in Supplementary section 7.3

* BSE= Backscattered Electrons, SE= Secondary Electrons.

7.3 Supplementary Figures 16-27: SEM images of AuBP@UIO-66

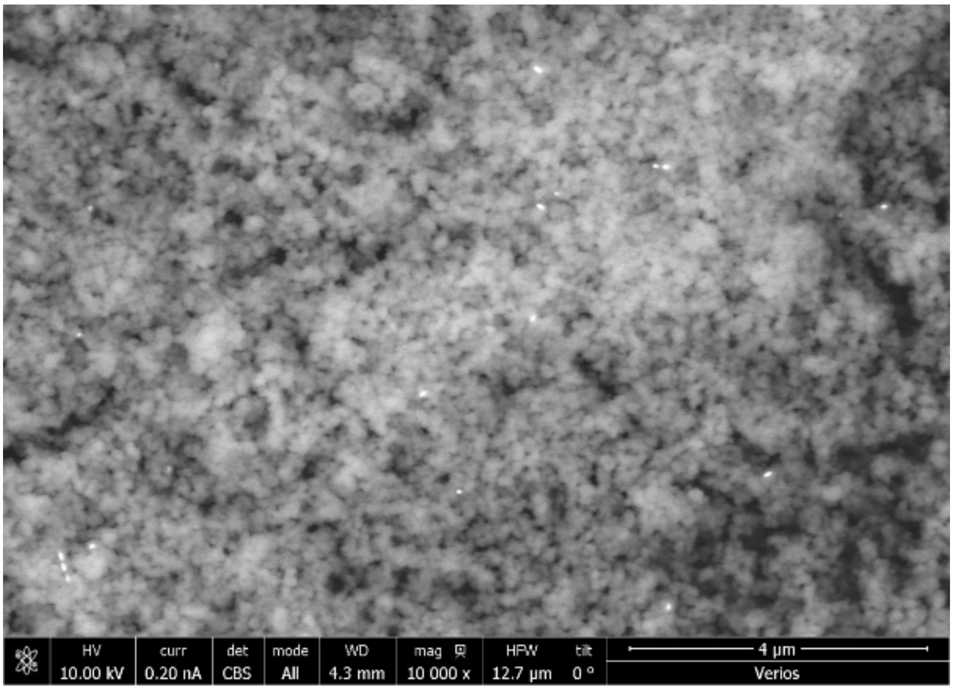

Supplementary figure 16 | SEM image a, entry 1, supplementary table 3.

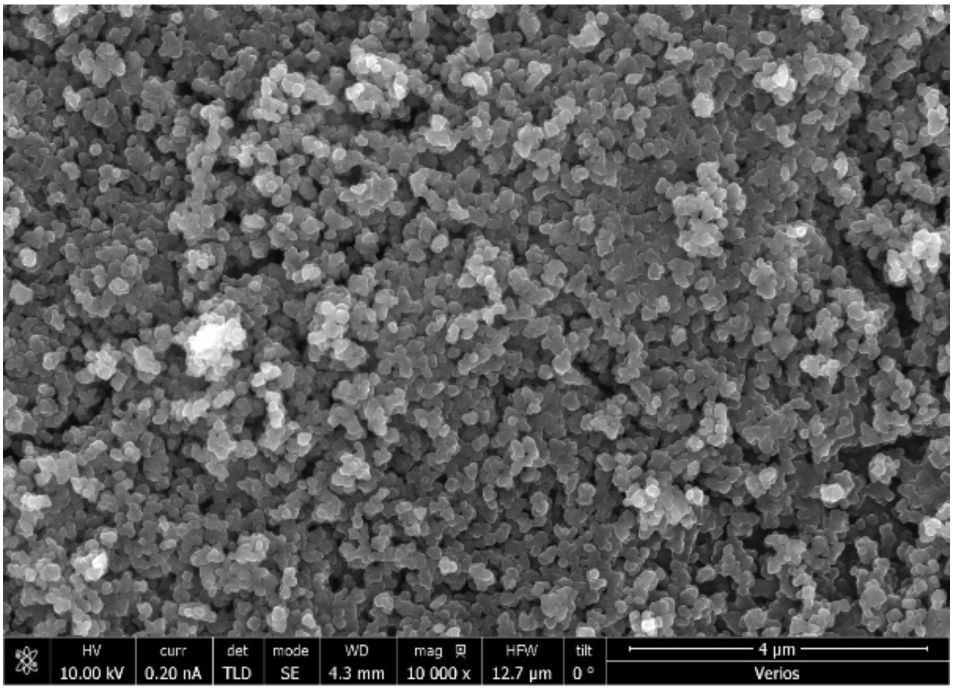

Supplementary figure 17 | SEM image a, entry 2, supplementary table 3.

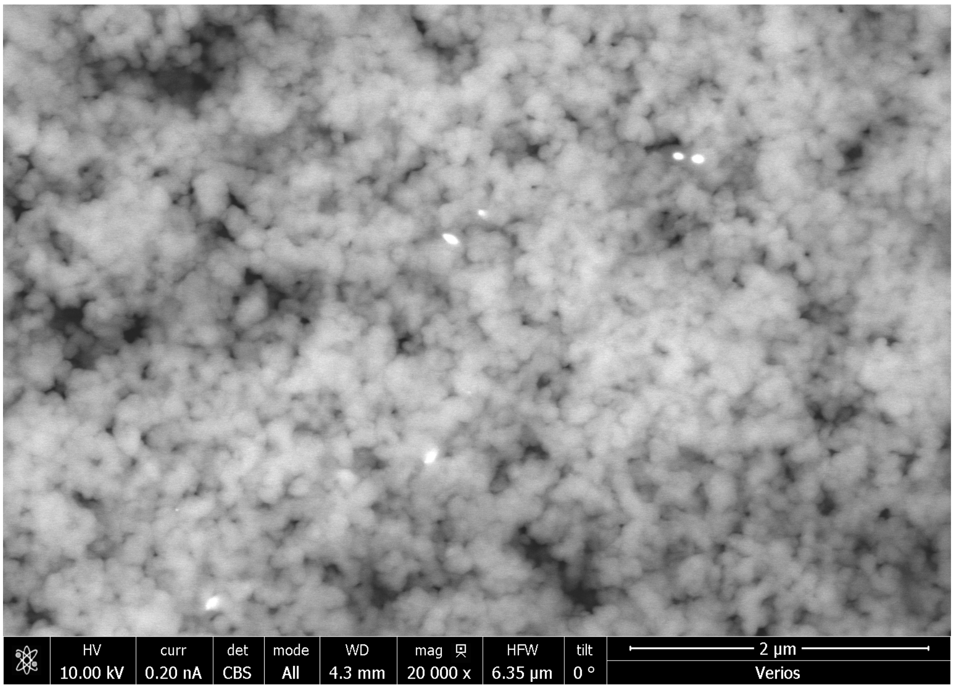

Supplementary figure 18 | SEM image b, entry 3, supplementary table 3.

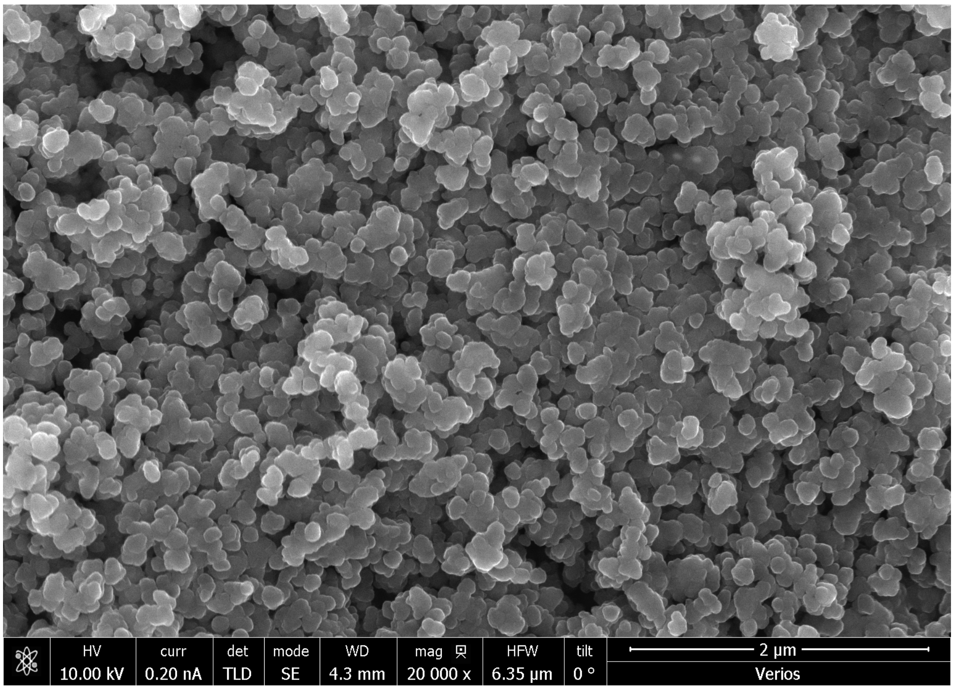

Supplementary figure 19 | SEM image b, entry 4, supplementary table 3.

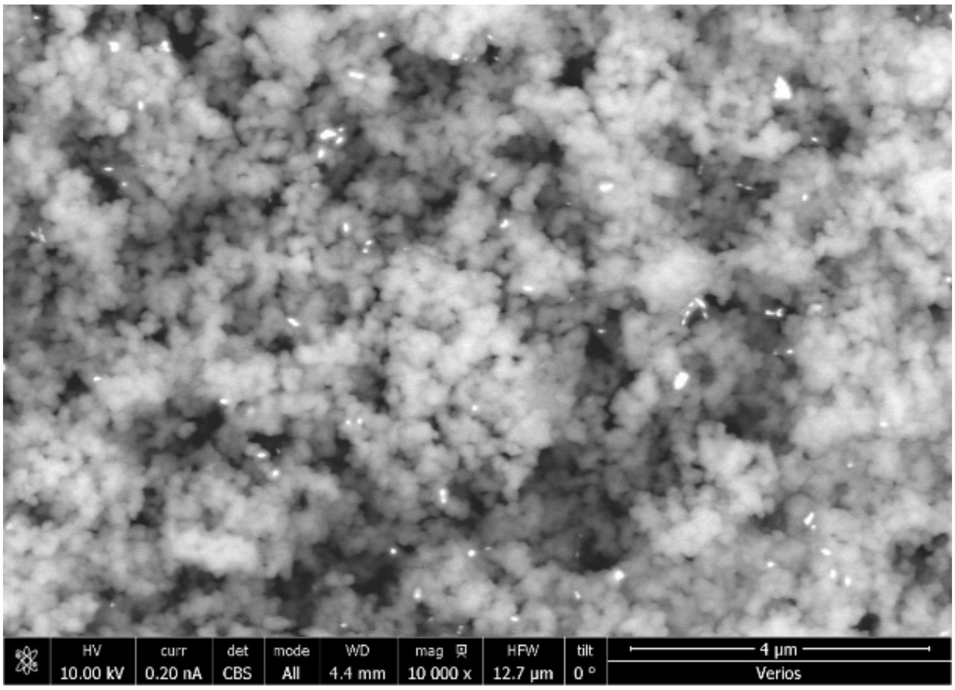

Supplementary figure 20 | SEM image c, entry 5, supplementary table 3.

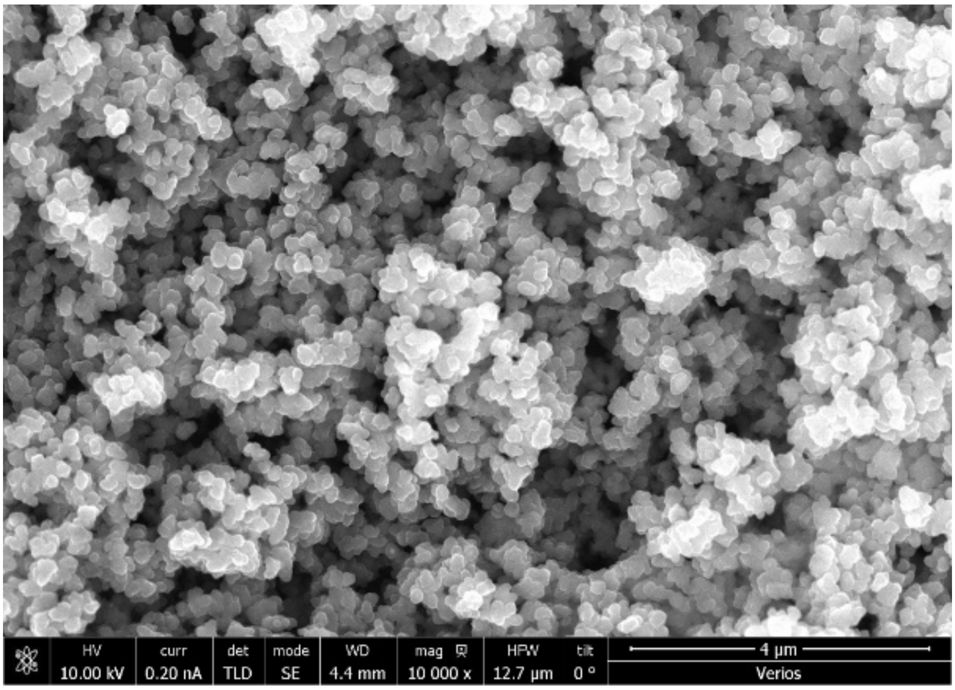

Supplementary figure 21 | SEM image c, entry 6, supplementary table 3.

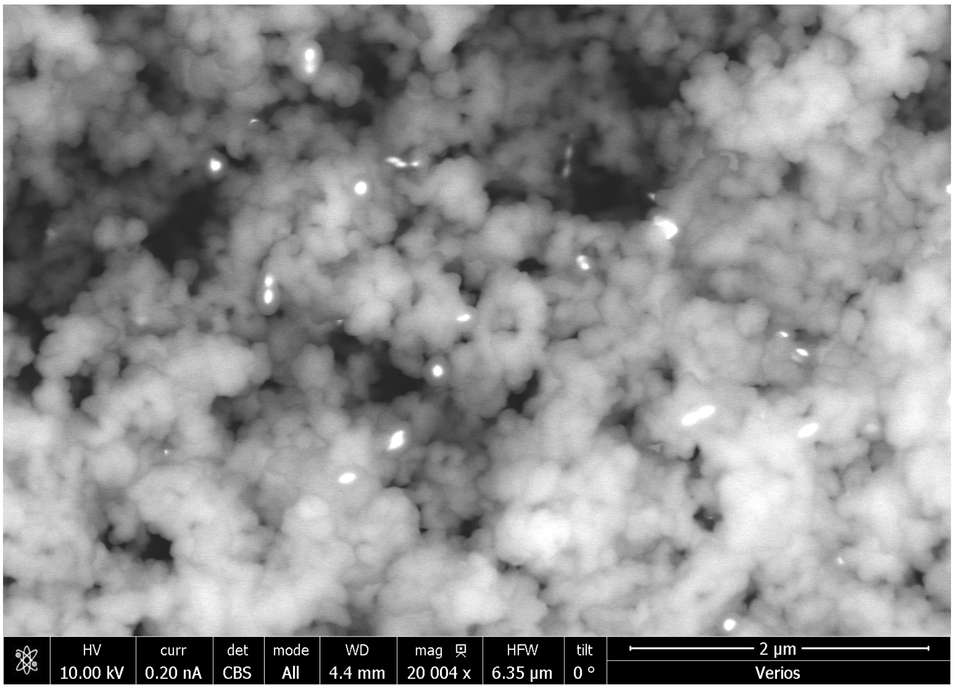

Supplementary figure 22 | SEM image d, entry 7, supplementary table 3.

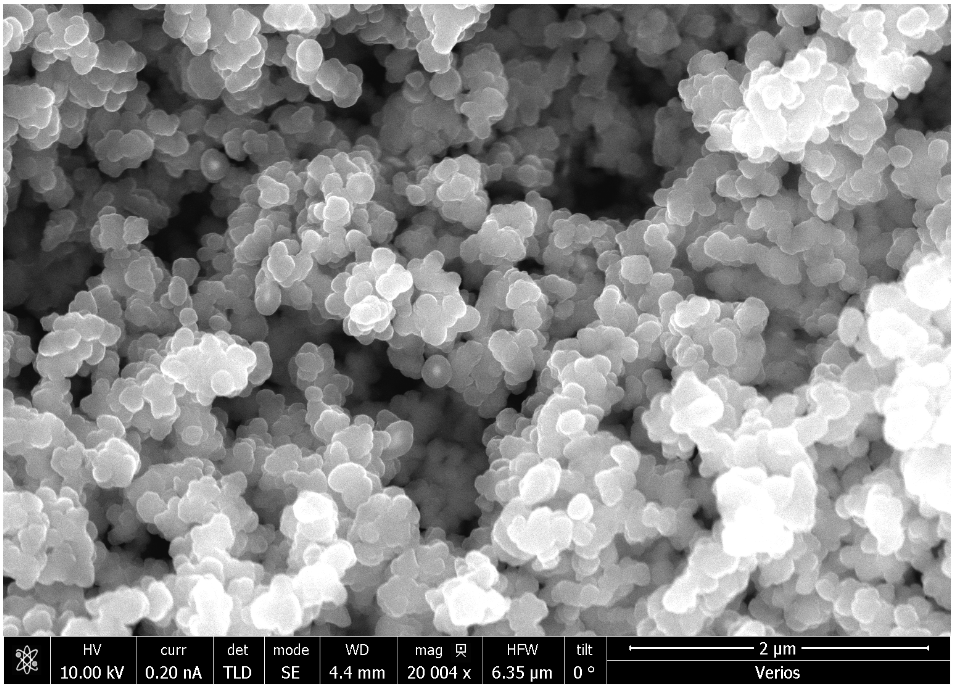

Supplementary figure 23 | SEM image d, entry 8, supplementary table 3.

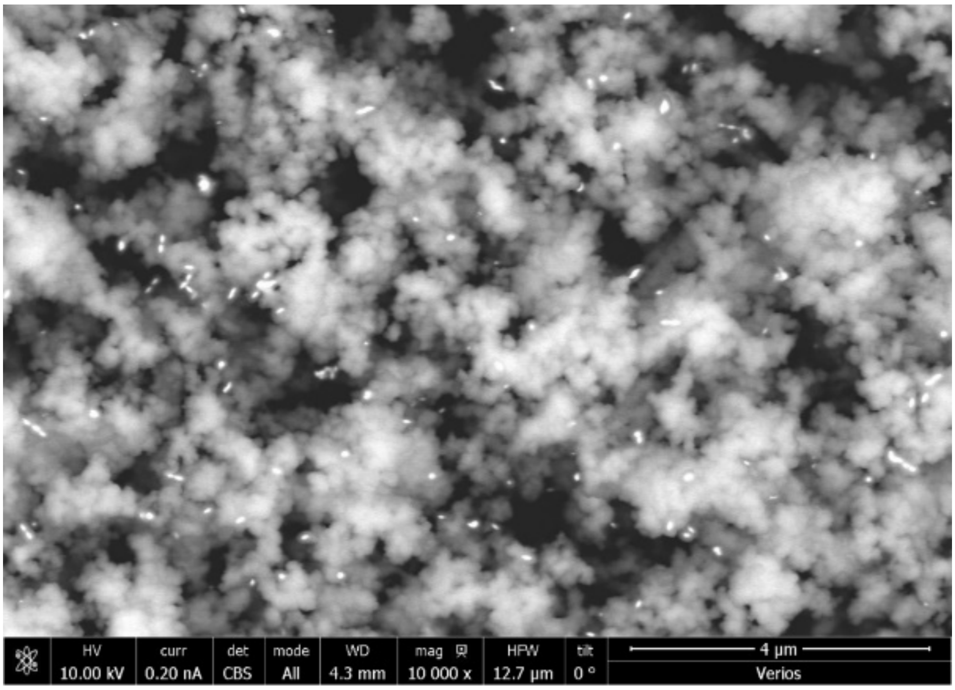

Supplementary figure 24 | SEM image e, entry 9, supplementary table 3.

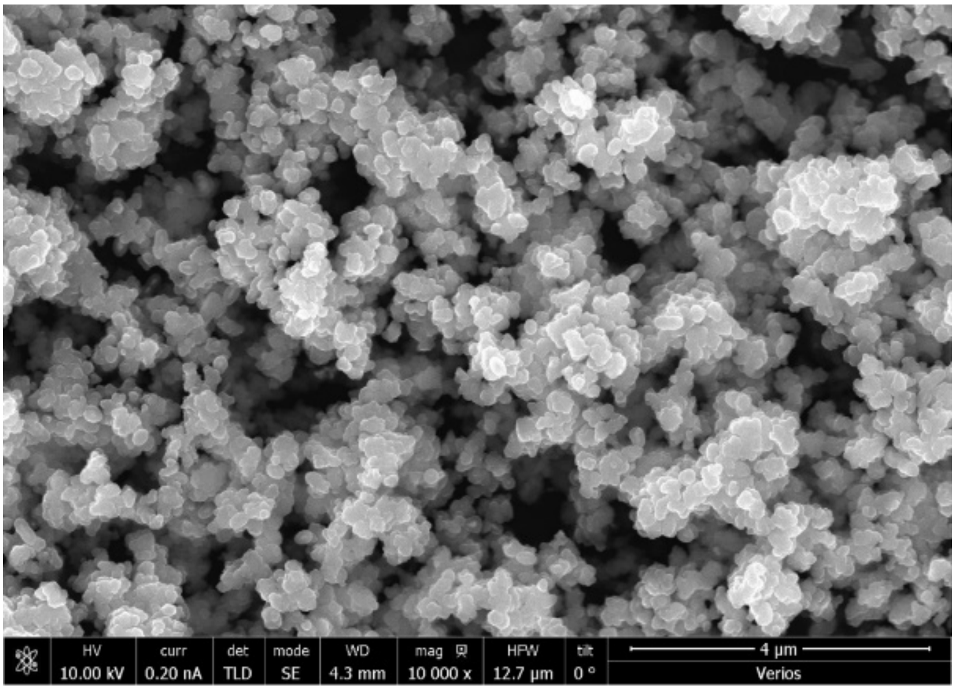

Supplementary figure 25 | SEM image e, entry 10, supplementary table 3.

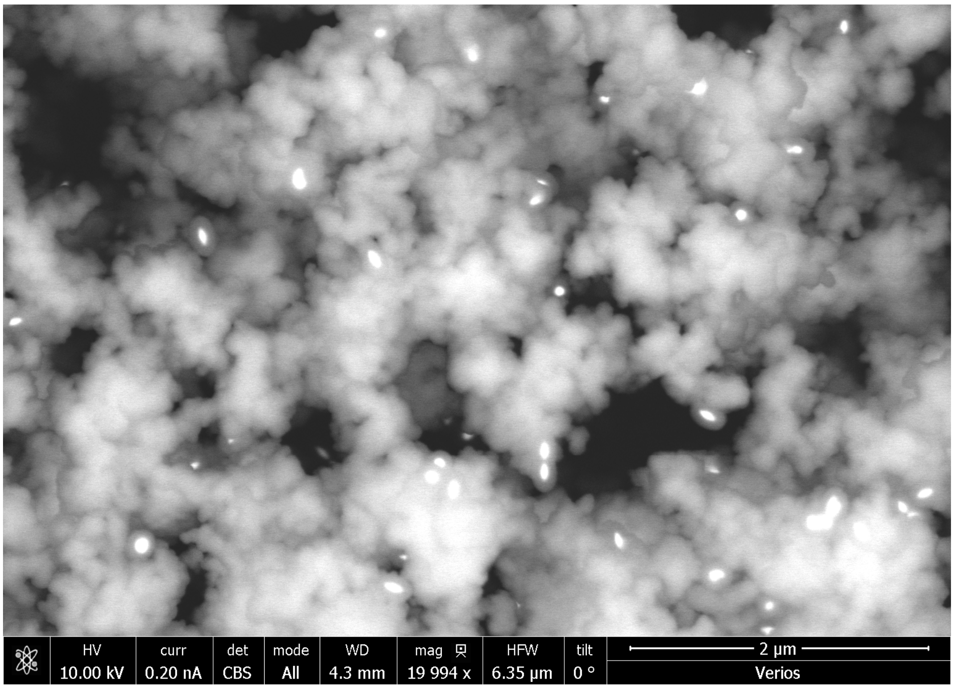

Supplementary figure 26 | SEM image f, entry 11, supplementary table 3.

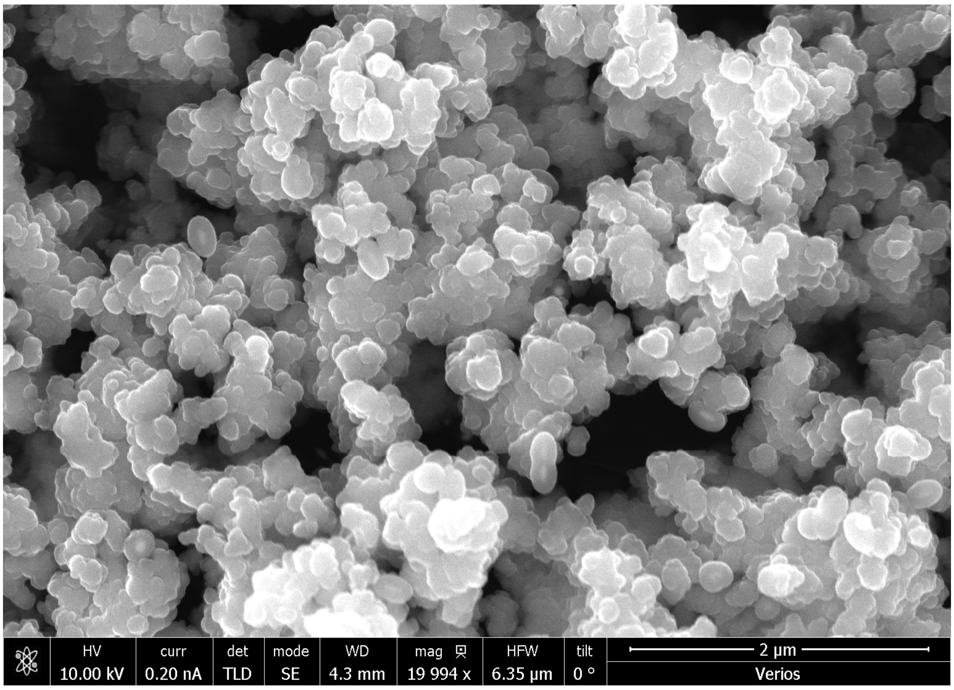

Supplementary figure 27 | SEM image f, entry 12, supplementary table 3.

7.4 Supplementary Table 4: ICP-OES of AuBPs@UIO-66

Supplementary table 4

Sample	Synthesis temperature	Au 242.795 (mg/l)	Au 267.595 (mg/l)	Au 174.050 (mg/l)	Au 197.819 (mg/l)	Au 201.265 (mg/l)	Average (mg/l)	SD (mg/l)
Blank	-	0.065	0.096	< -0.232	< 0.085	< 0.049	0.0805	0.0155
1	60 °C	1.542	1.538	1.281	1.509	1.534	1.4808	0.1006
2	80 °C	1.854	1.913	1.582	1.898	1.957	1.8408	0.1335
3	100 °C	2.734	2.758	2.435	2.732	2.81	2.6938	0.1324

ICP-OES results of different UIO-66

7.5 Supplementary Figures 28-30: TEM images of AuBP@UIO-66

Supplementary figure 28 | TEM image of $AuBPs_{850}$@UIO-66 synthesized at 100 °C. $AuBPs@SiO_2$ highlighted in red.

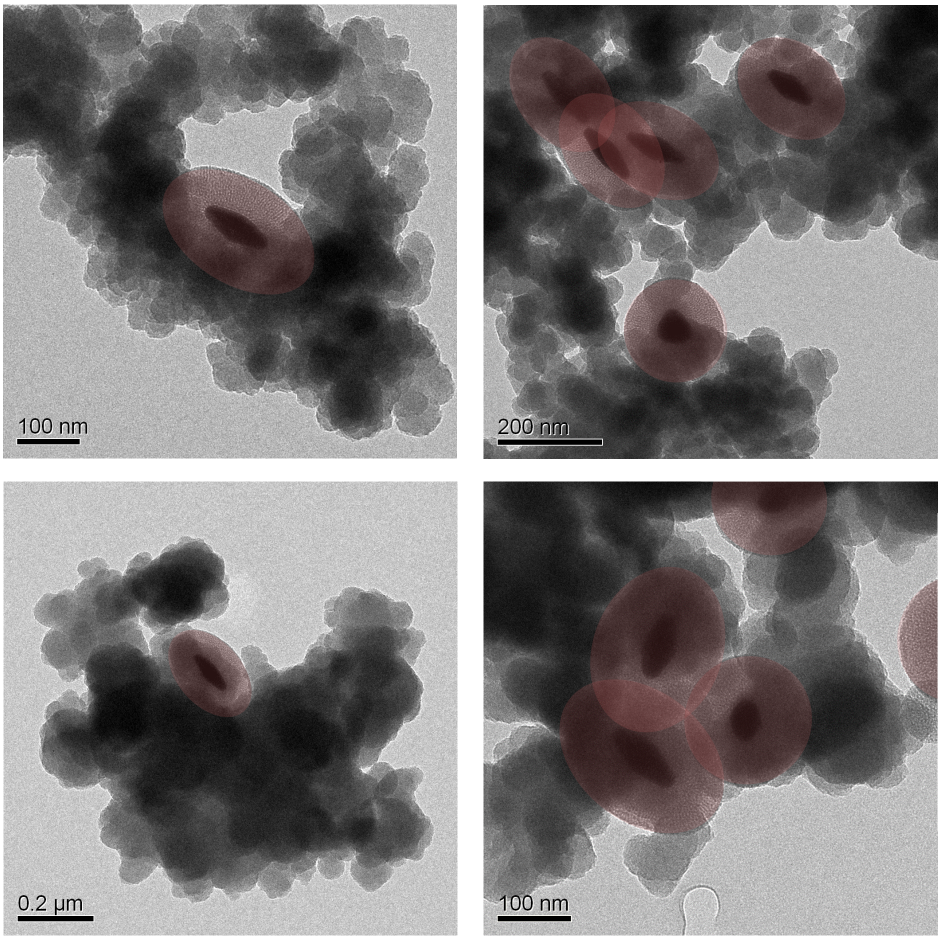

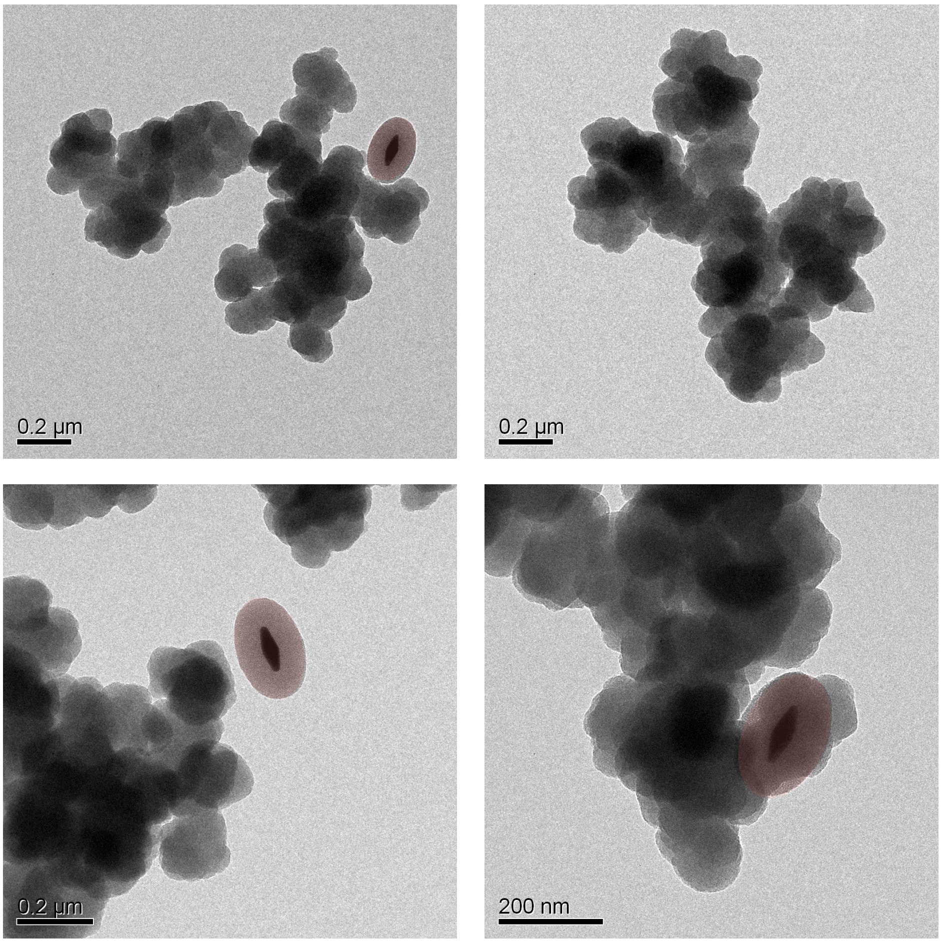

Supplementary figure 29 | TEM image of $AuBPs_{850}$@UIO-66 synthesized at 80 °C. AuBPs@SiO_2 highlighted in red.

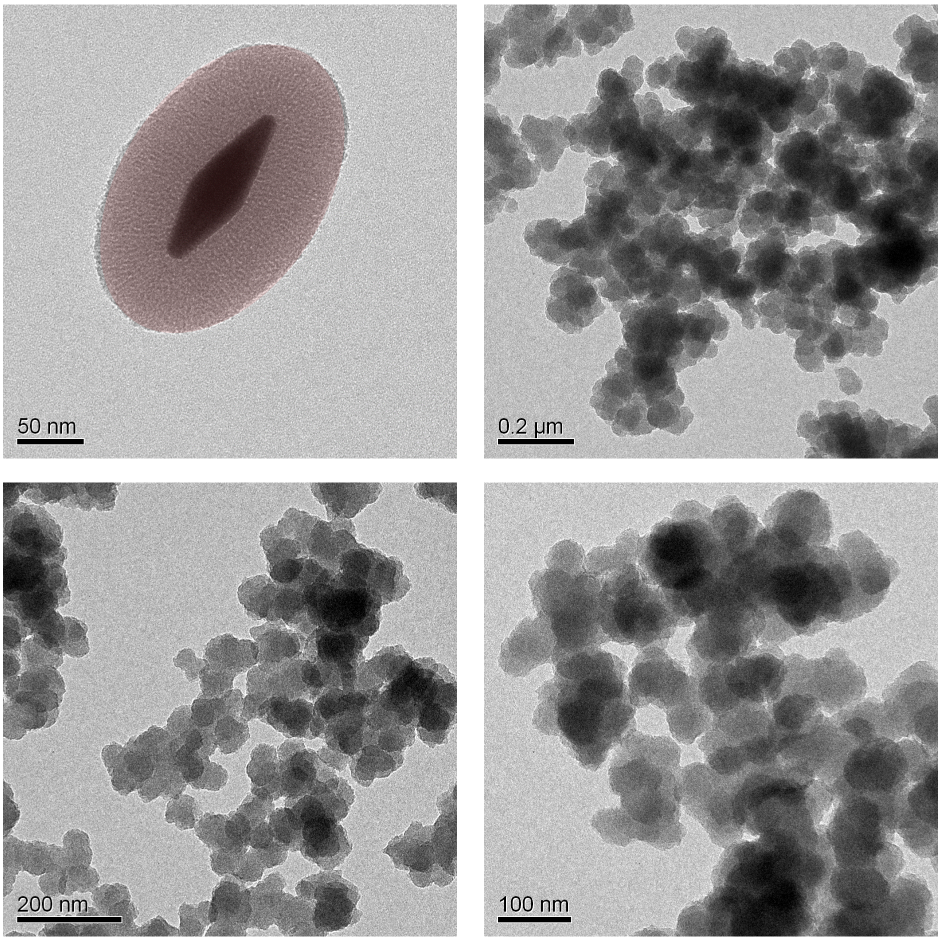

Supplementary figure 30 | TEM image of $AuBPs_{850}$@UIO-66 synthesized at 60 °C. AuBPs@SiO_2 highlighted in red.

7.6 Supplementary Table 5: Surface areas of AuBP@UIO-66

Supplementary table 5

Entry	Temperature (°C)	Surface area (m^2/gr)
1	60	1530
2	80	1660
3	100	1553
4	120	736
5	Katz *et al.*[1]	1580
6	Cavka *et al.*[2]	1187

Surface areas of different UIO-66

7.7 Supplementary Figure 31-33: Size distribution of UIO-66 particles

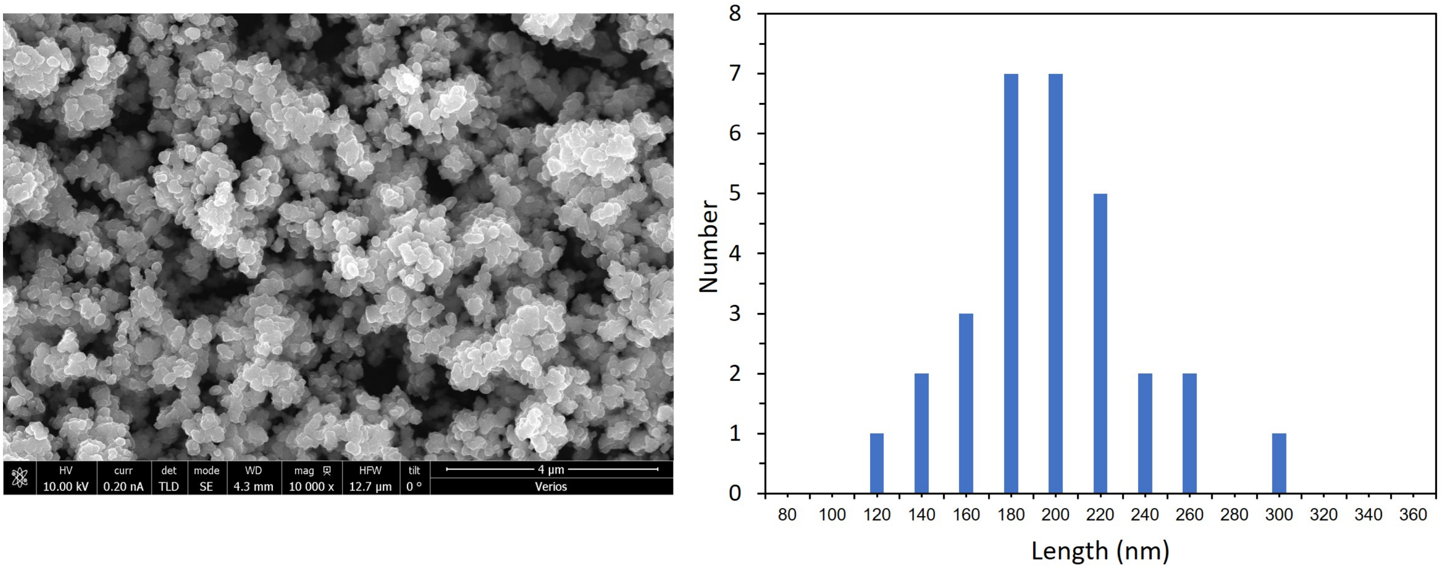

Supplementary figure 31 | (left) SEM image of AuBPs@UIO-66 synthesized at 100 °C photothermally. (right) Size distribution measurement according to the SEM image.

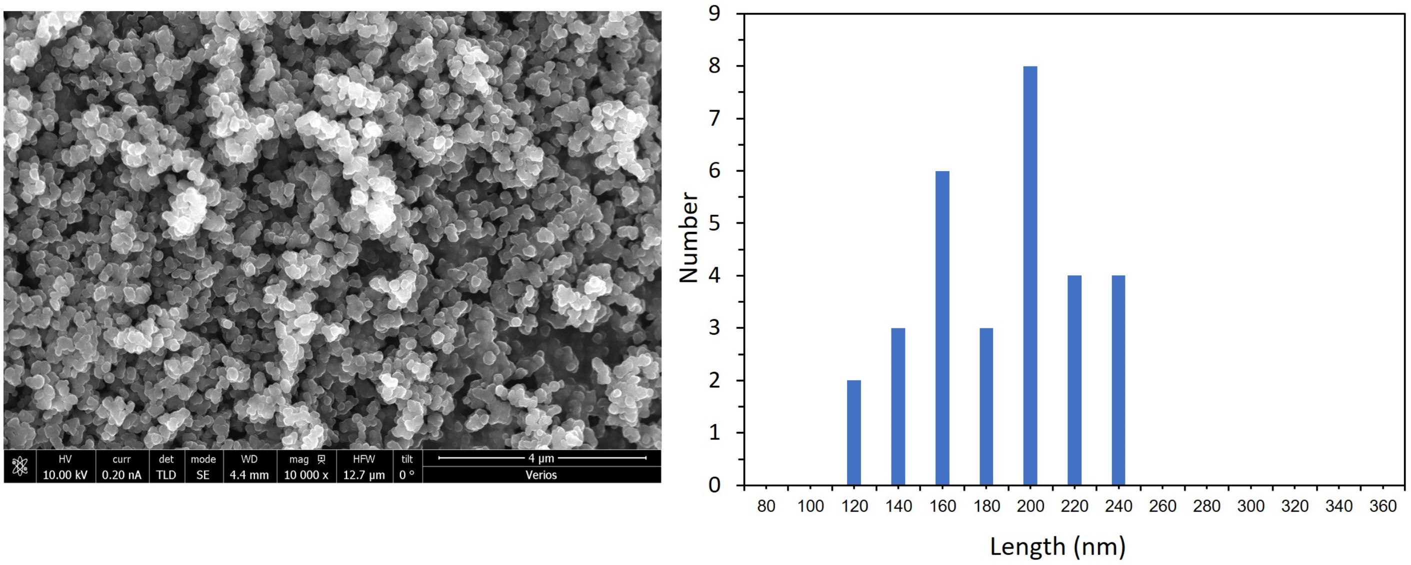

Supplementary figure 32 | (left) SEM image of AuBPs@UIO-66 synthesized at 80 °C photothermally. (right) Size distribution measurement according to the SEM image.

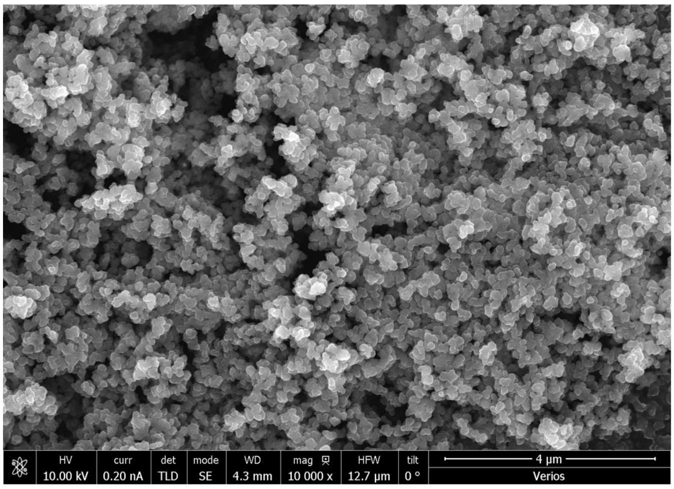

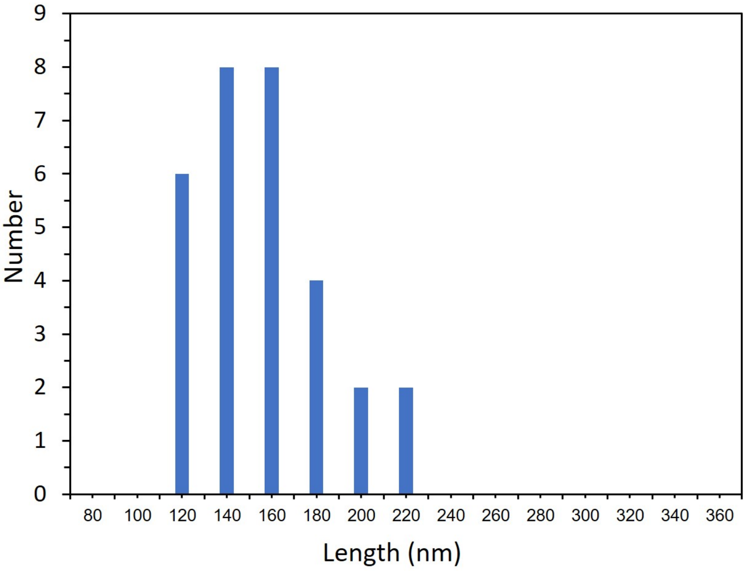

Supplementary figure 33 | (left) SEM image of AuBPs@UIO-66 synthesized at 60 °C photothermally. (right) Size distribution measurement according to the SEM image.

8. Supplementary figure 34: Zeta potential

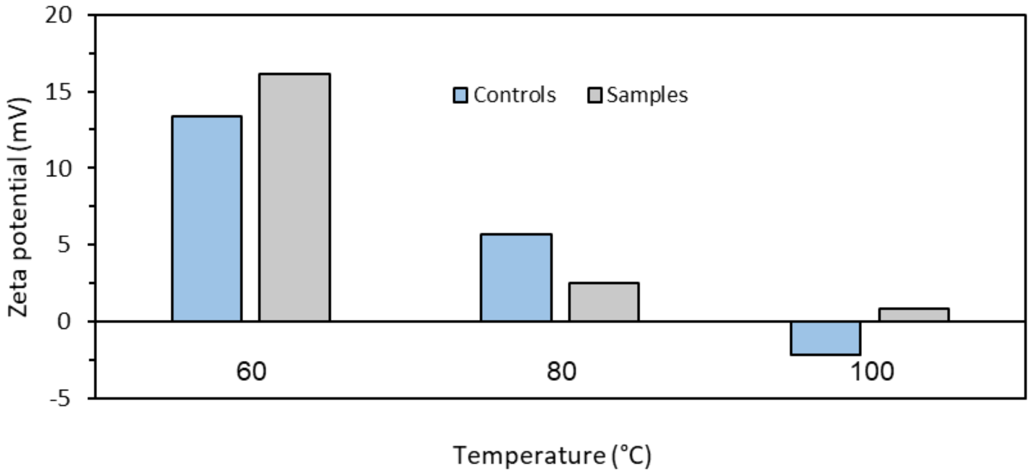

Supplementary figure 34 | Zeta potential results of samples of AuBPs that were heated in presence of BDC, to different temperatures. The controls excluded BDC.

9. Supplementary Note 7: AuBPs@SiO_2 and zirconium interaction

9.1 Supplementary figure 35-37: AuBPs with $ZrCl_4$ at different temperatures

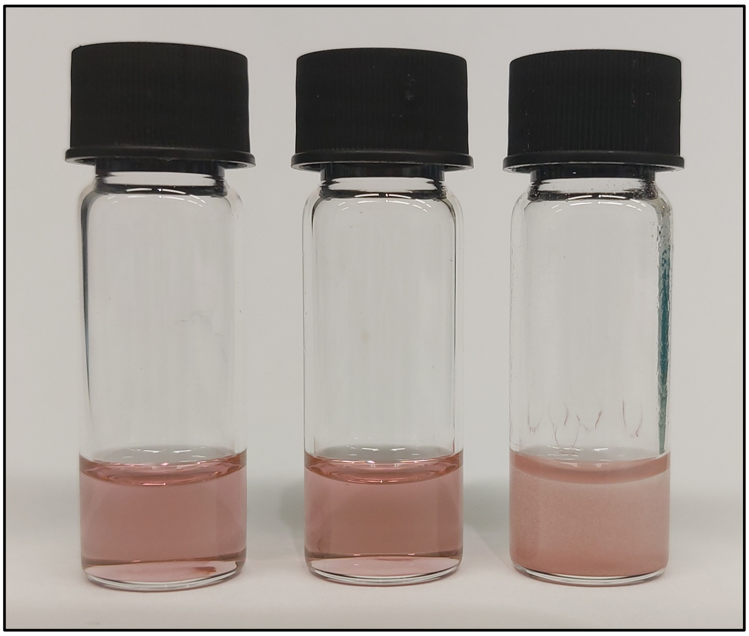

Supplementary figure 35 | Image of the vails of solution contains AuBPs (2 OD) and $ZrCl_4$ in DMF and HCl after heated to different temperatures (From left: 60 °C, 80 °C and 100 °C)

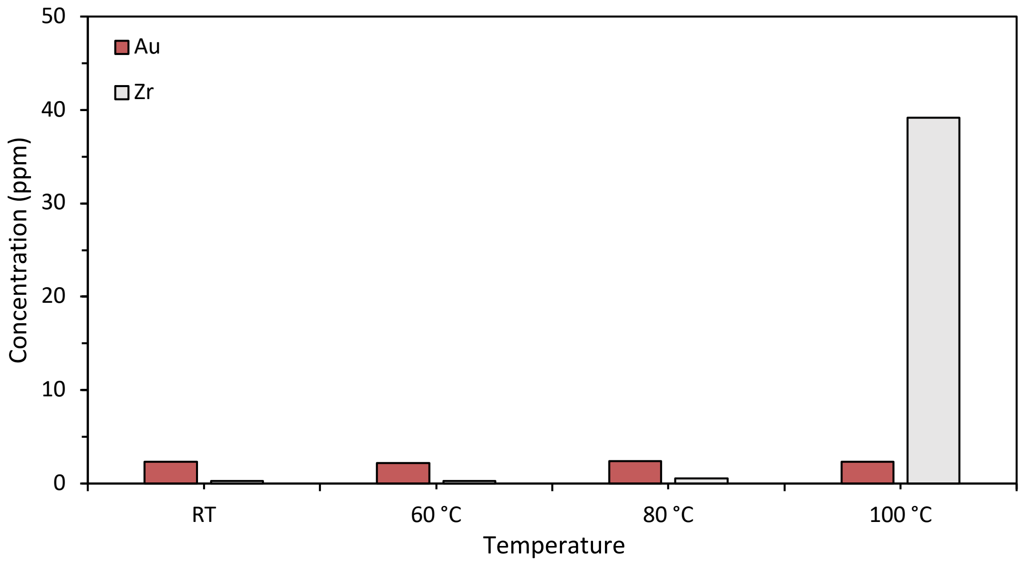

Supplementary figure 36 | ICP-OES Results of AuBPs Heated with $ZrCl_4$ at Different Temperatures photothermally.

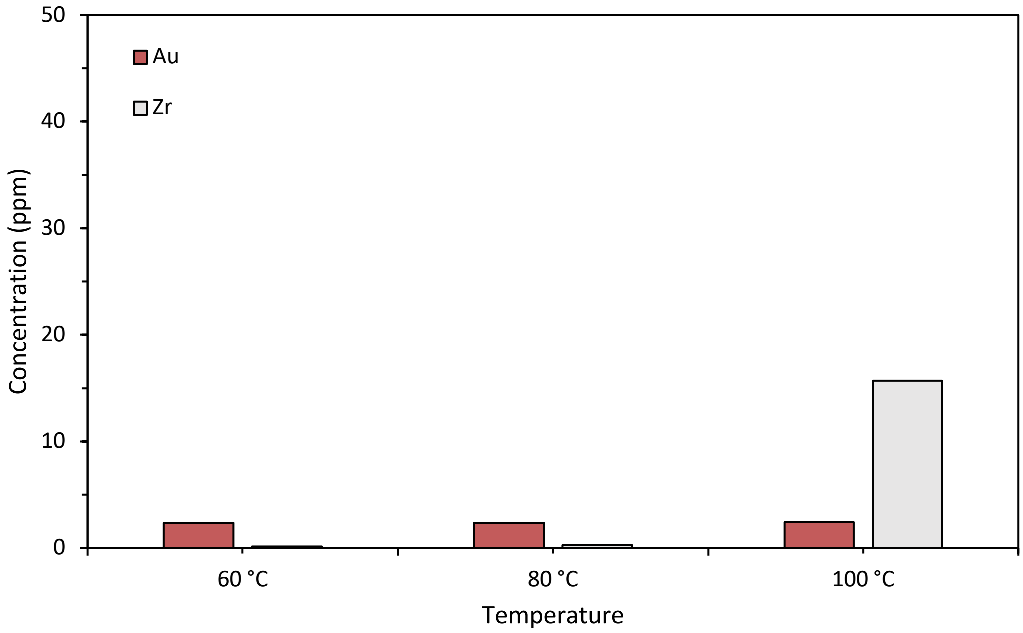

Supplementary figure 37 | ICP-OES Results of AuBPs Heated with $ZrCl_4$ at Different Temperatures conventionally.

9.2 Supplementary Figure 38-40: STEM images and EDS analysis

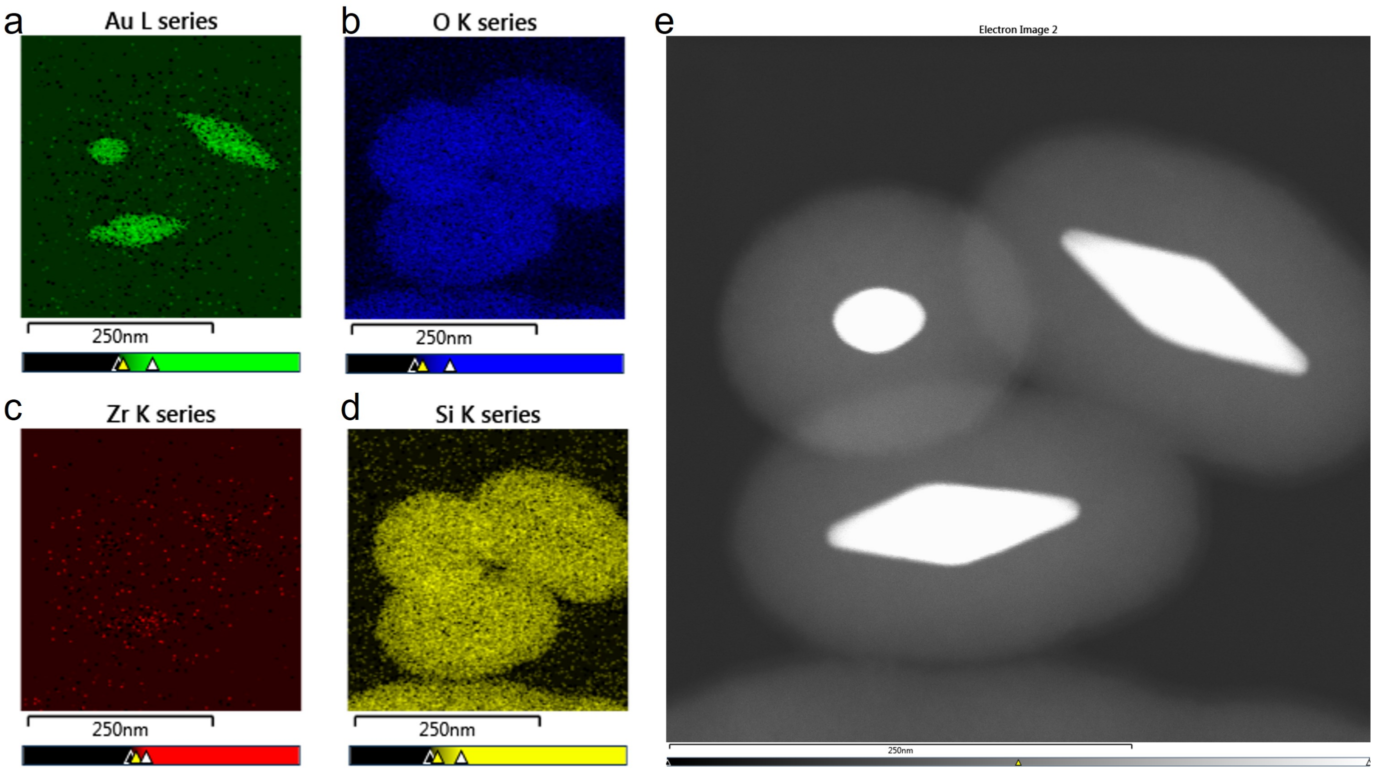

Supplementary figure 38 | EDS elemental mapping of AuBPs that were heated with $ZrCl_4$ at 60 °C **a,** Au L series. **b,** O K series. **c,** Zr K series. **d,** Si K series. **e,** STEM image.

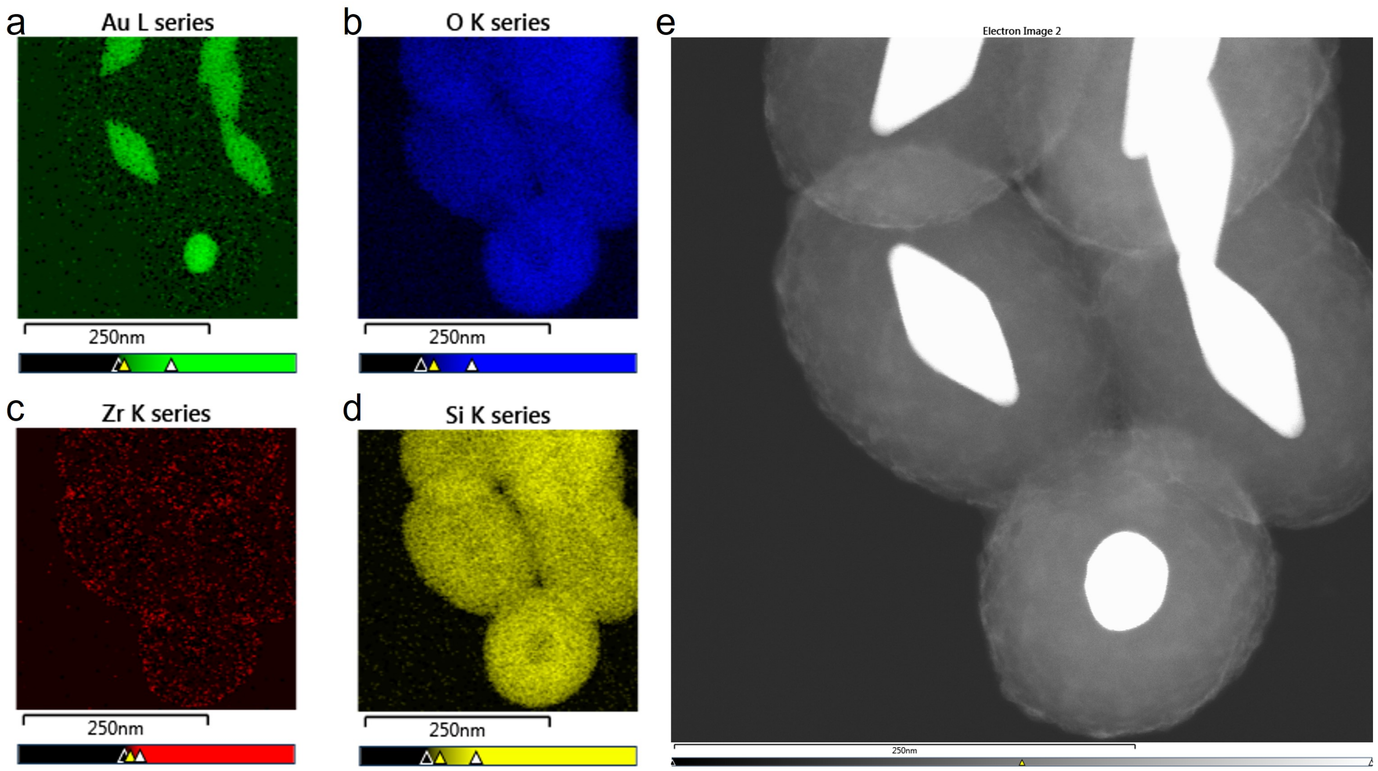

Supplementary figure 39 | EDS elemental mapping of AuBPs that were heated with $ZrCl_4$ at 80 °C **a,** Au L series. **b,** O K series. **c,** Zr K series. **d,** Si K series. **e,** STEM image.

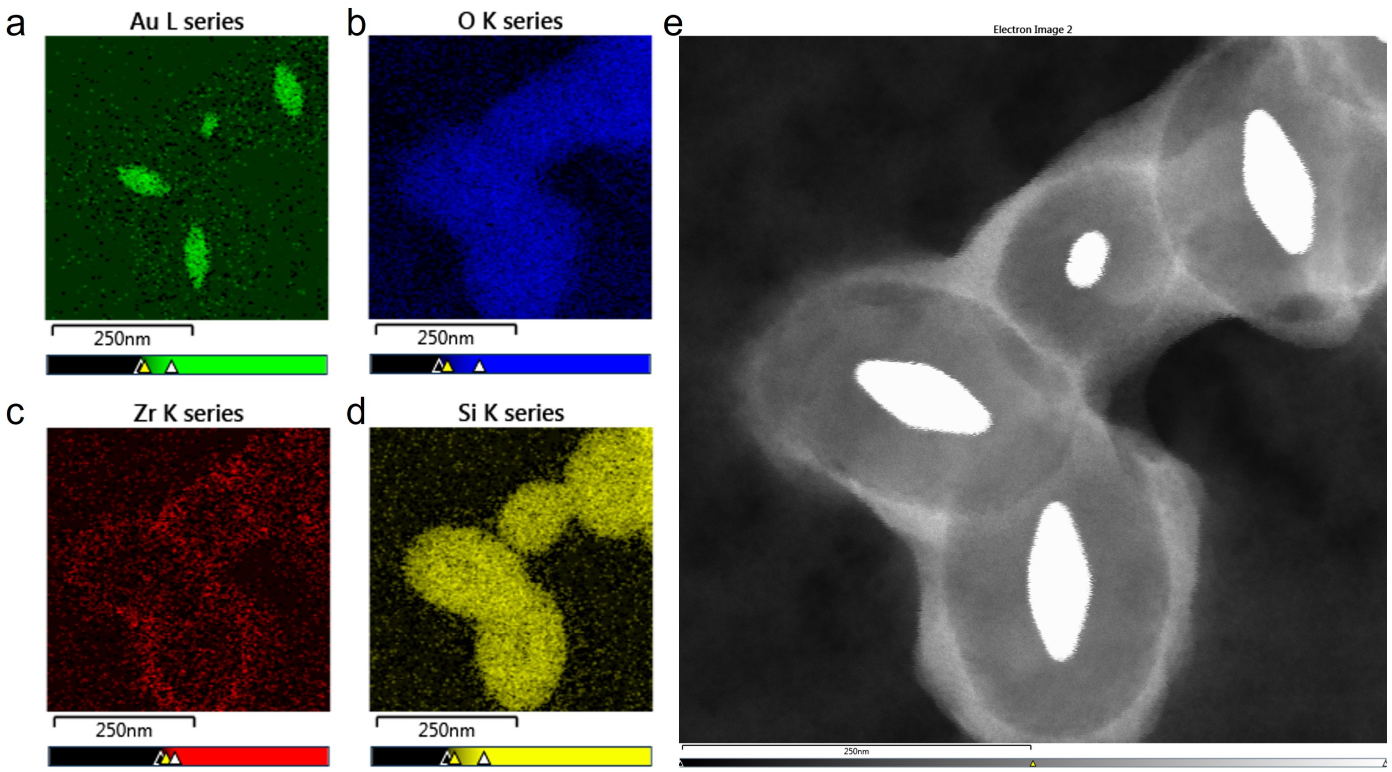

Supplementary figure 40 | EDS elemental mapping of AuBPs that were heated with $ZrCl_4$ at 100 °C **a,** Au L series. **b,** O K series. **c,** Zr K series. **d,** Si K series. **e,** STEM image.

9.3 Supplementary Figure 41: X-ray photoelectron spectroscopy

In an acidic environment (pH 0-2), two main Zr(IV) species coexist: $[ZrOH]^{3+}$ and $[Zr_4(OH)_8(H_2O)_{16}]^{8+}$ tetramer.[3] As the temperature rises, the tetramer undergoes condensation, forming the $[Zr_8(OH)_{20}(H_2O)_{24}]^{12+}$ octamer, [4] with further temperature increase leading to topotactic dehydration, creating amorphous zirconium oxide/oxyhydroxide species, $ZrOx(OH)_{4-2x}$. [5]

This is demonstrated nicely by deconvolution of the high resolution XPS spectra of the O 1*s* regions at various temperatures. At 60 °C, the O 1*s* spectrum features two main binding energies to a heteroatom (X = Si, Zr) corresponding to adsorbed/structural water (X-OH_2, 532.21 eV) and structural hydroxide (X-OH, 531.19 eV) species with a percentage ratio of 58.0 : 42.0, respectively (Supplementary Fig. 41a).

In comparison, at 100 °C the O 1*s* spectrum features three main binding energies, corresponding to adsorbed/structural water (M-OH_2, 532.89 eV), structural hydroxide (M-OH, 531.07 eV) and structural oxide (M-O-M, 529.34 eV) species with a percentage ratio of 33.3 : 52.5 : 14.2, respectively (Supplementary Fig. 41b). The presence of the binding energy peak associated with structural oxide in the XPS spectra of the sample heated at 100 °C, and its absence in the spectra of the 60 °C sample, indicates the formation of a zirconium oxide/oxyhydroxide layer in the sample exposed to the higher temperature.

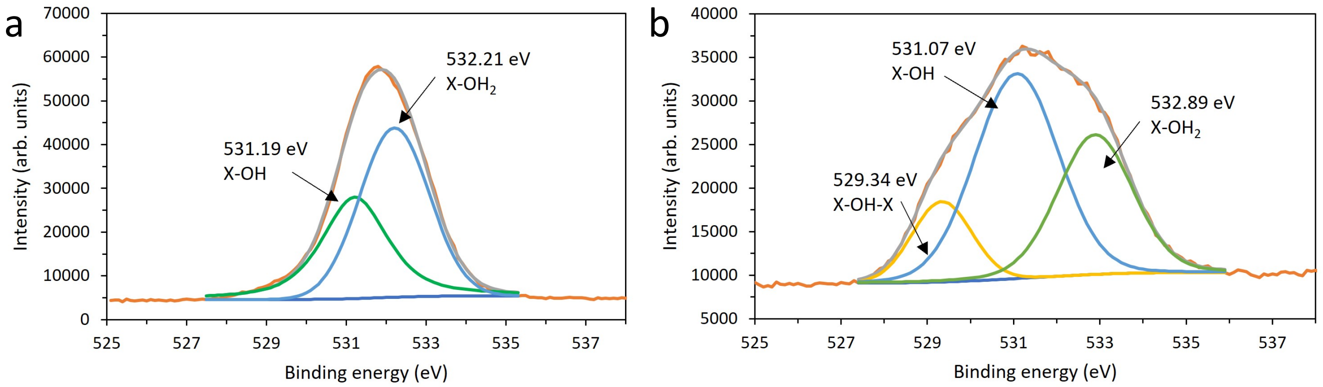

Supplementary figure 41 | XPS spectra of O 1*s* region of AuBP@Zr that were heated to 60 °C (a), and 100 °C (b). X= Zr, Si.

9.4 Supplementary Figure 42: PXRD pattern of AuBPs covered with zirconium oxide.

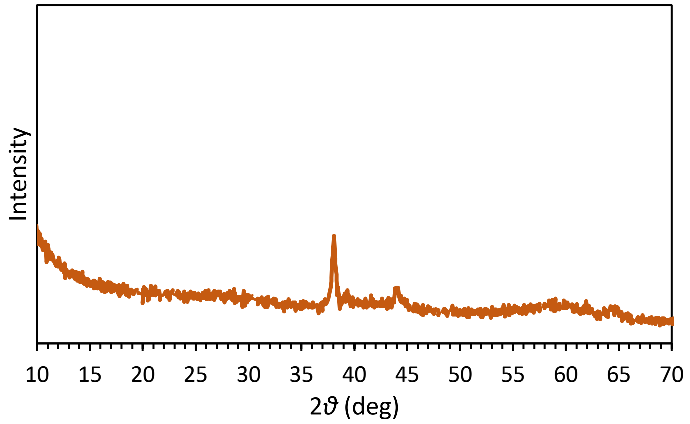

Supplementary Figure 42 | PXRD pattern of AuBPs that heated to 100 °C in presence of $ZrCl_4$. The peaks shown in the pattern correspond to the AuBPs.[6] The absence of additional peaks suggest that the zirconium oxide layer is amorphous.

9.5 Supplementary Figure 43-44: TEM images of AuBPs@Zr@UIO-66

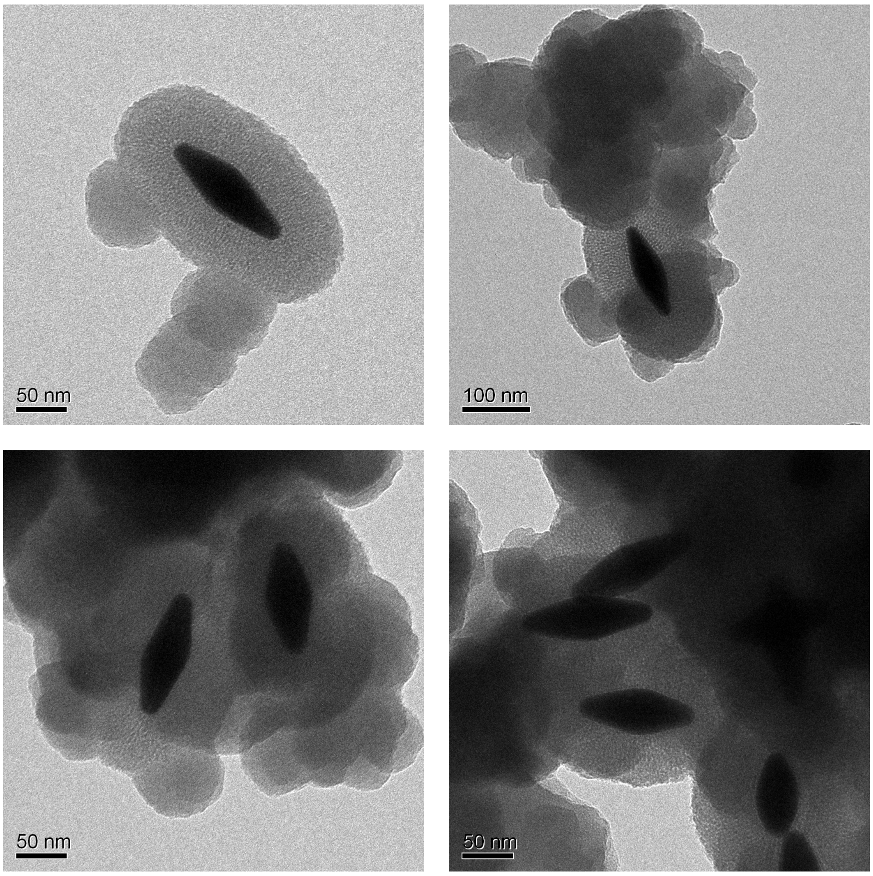

Supplementary figure 43 | TEM Images of AuBP@ UIO-66 synthesized with AuBPs pre-coated with zirconium at 100°C 2 OD.

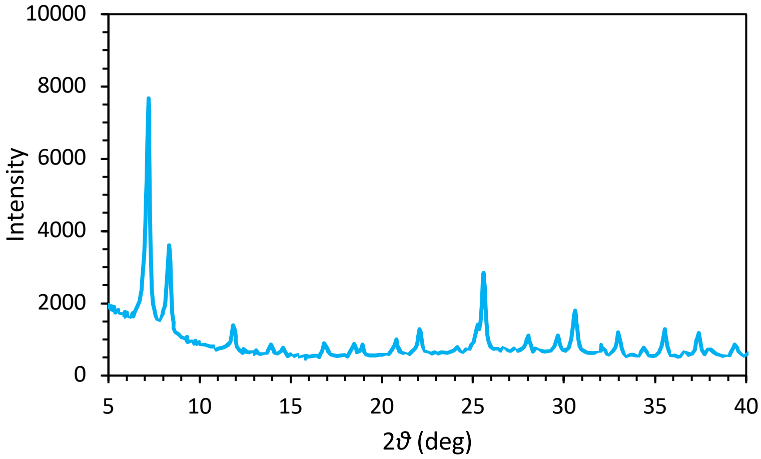

Supplementary figure 44 | PXRD pattern of the product from supplementary figure 43

10. Supplementary Figure 45-46: UIO-66 synthesis using methylene blue

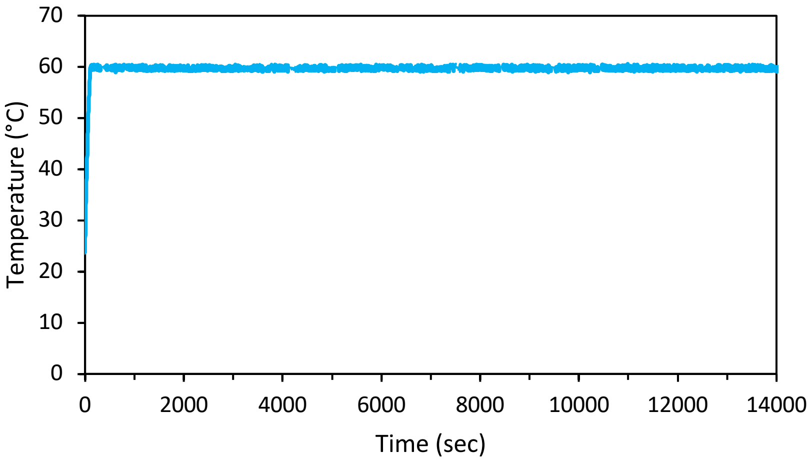

Supplementary figure 45 | Temperature profile of photothermal synthesis of UIO-66 using methylene blue (2 OD). Using 660 nm 100 W LED.

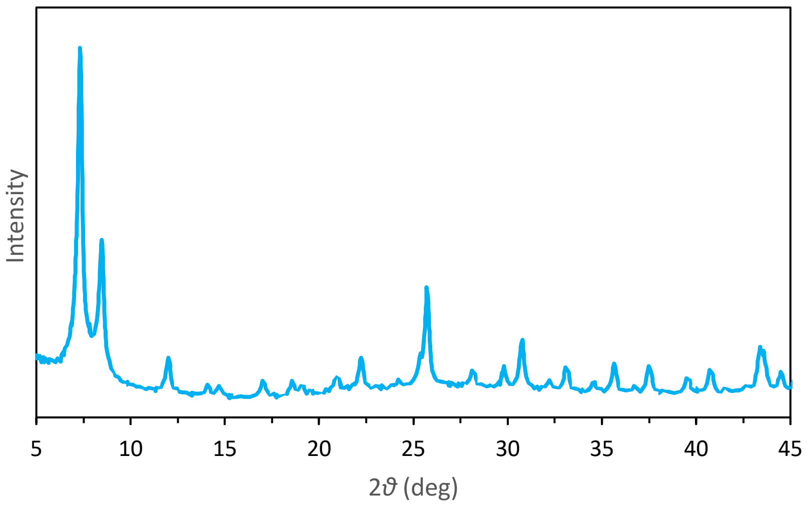

Supplementary figure 46 | PXRD pattern of UIO-66 synthesized photothermally, using methylene blue.

11. Supplementary Figure 47-48: Conventional synthesis of UIO-66

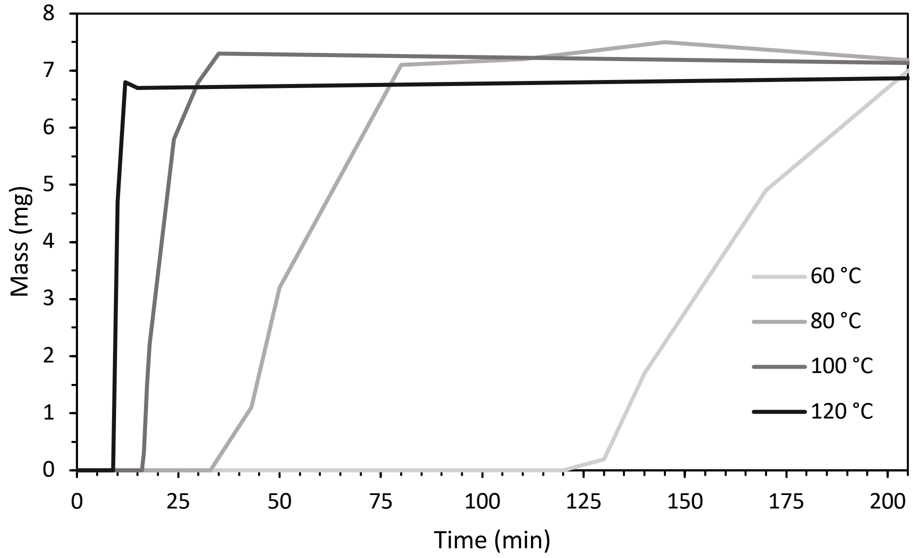

Supplementary figure 47 | Rate of conventional synthesis of UIO-66

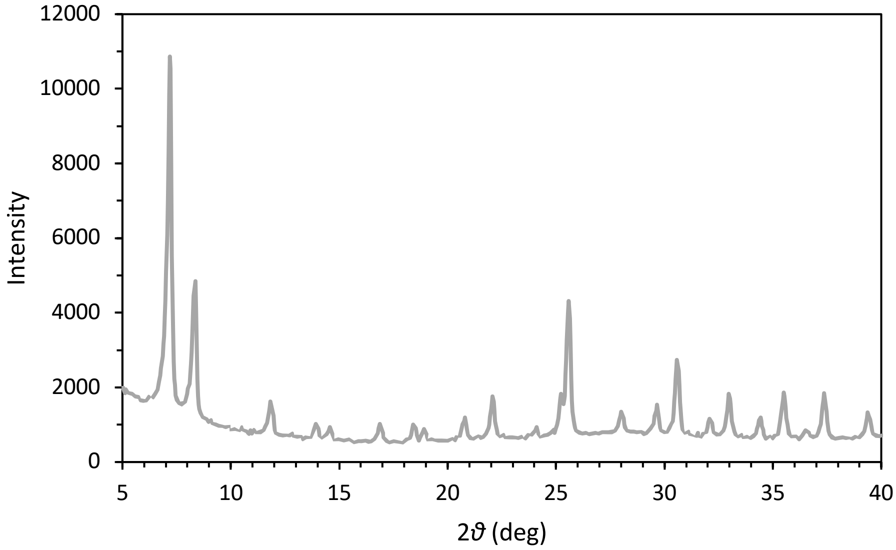

Supplementary figure 48 | PXRD pattern of UIO-66 conventionally synthesized at 100 °C for 20 minutes.

12. Supplementary Note 8: $AuBP_{850}$ recycling

<u>Procedure</u>

UIO-66 reaction was prepared as described in section 4.2, modified to volume of 2 ml, with $AuBP_{850}$ concentration of 2 OD. Each cycle was completed with a 100W 850nm LED for 3 hours at 60 °C. After each cycle, the supernatant was separated from the product, then washed three time with ethanol. After drying, the AuBPs were added to a new UIO-66 reagents solution.

12.1 Supplementary Figure 49: Temperature profile

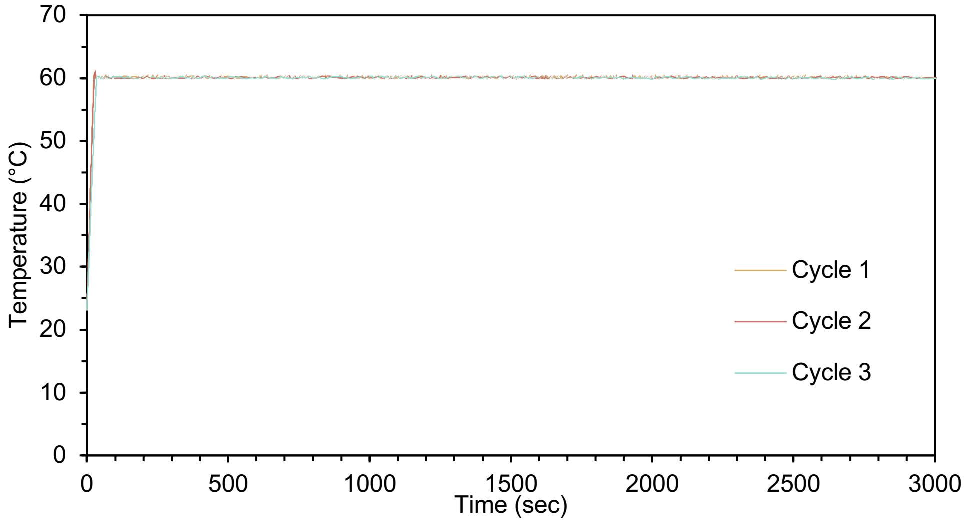

Supplementary figure 49 | Temperature profile of photothermal synthesis of UIO-66 using recycled AuBPs

12.2 Supplementary Table 6: UIO-66 mass

Supplementary table 6

Entry	Cycle	Mass (mg)
1	1	14.2
2	2	16
3	3	15.9

Masses of UIO-66 synthesized with recycled AuBPs

13. Supplementary Note 9: Versatility and scope of MOFs photothermal synthesis

13.1 Supplementary Figure 50-54: Photothermal synthesis using Carbon Black (CB)

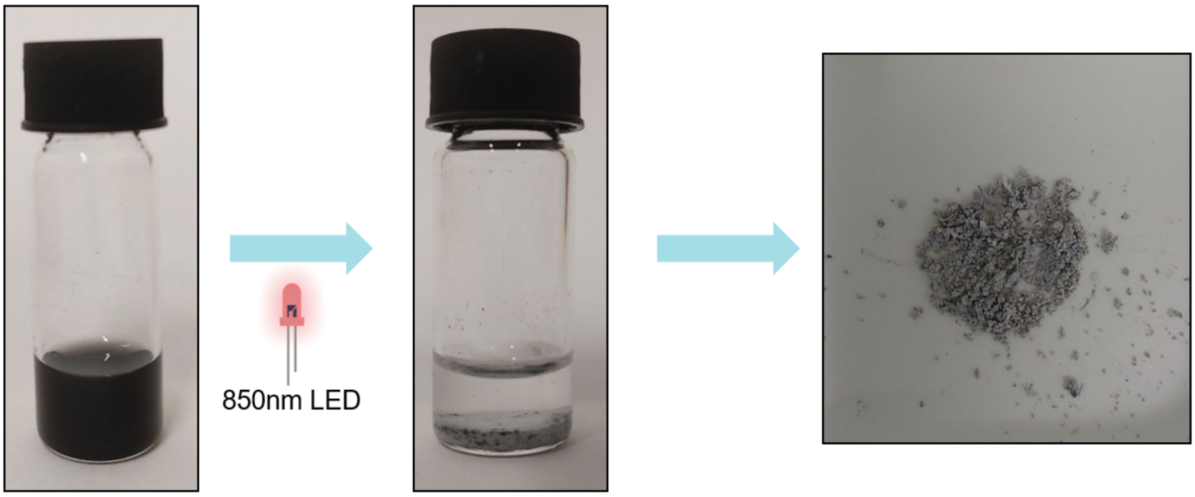

Supplementary figure 50 | Scheme of CB@UIO-66 synthesis using 850nm LED. 3 mg of CB, 1ml, 100 °C, 20 minutes.

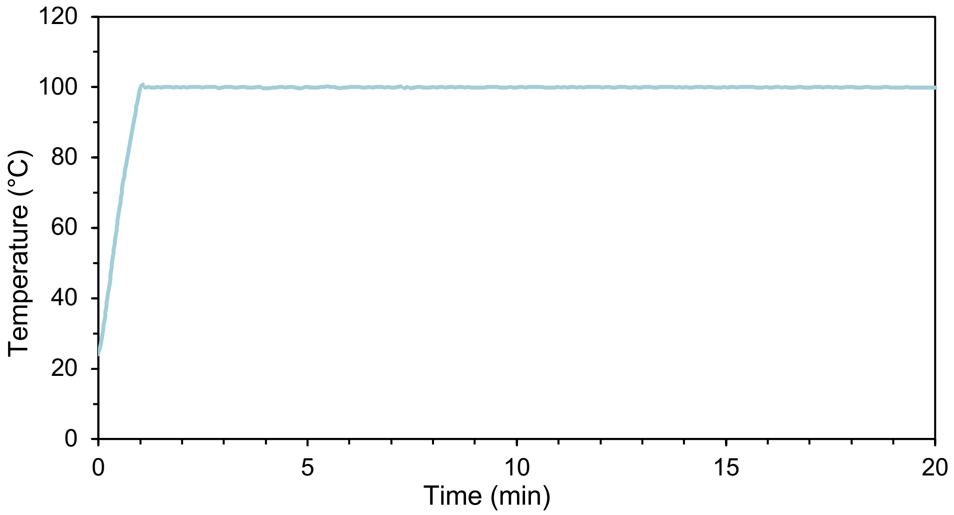

Supplementary figure 51 | Temperature profile of CB@UIO-66 synthesis using 850nm 100W LED

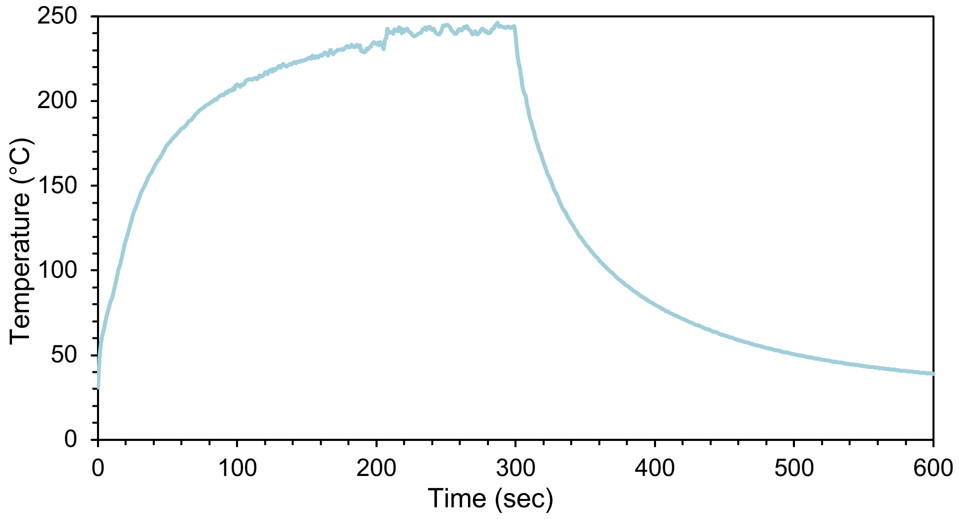

Supplementary figure 52 | Heating ability of CB@UIO-66 irradiated by 850nm 100W LED

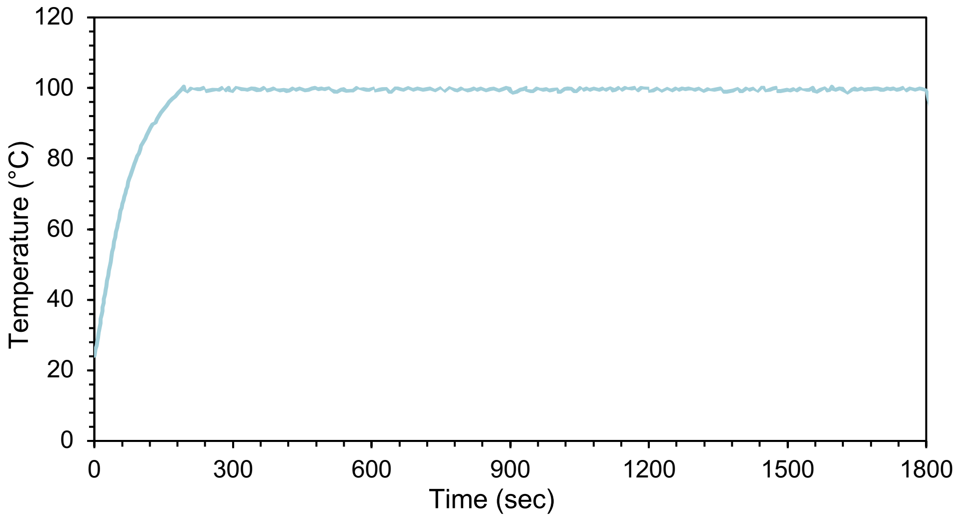

Supplementary figure 53 | Temperature profile of CB@UIO-66 synthesis using 520nm 100W LED

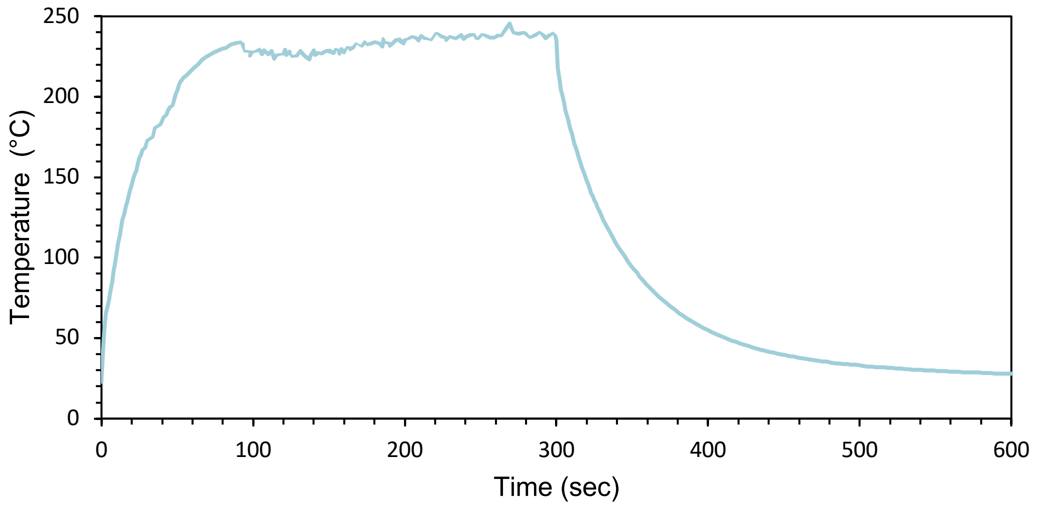

Supplementary figure 54 | Heating ability of CB@UIO-66 irradiated by 520nm 100W LED

13.2 Supplementary Figure 55-56: Photothermal synthesis using Activated charcoal (AC)

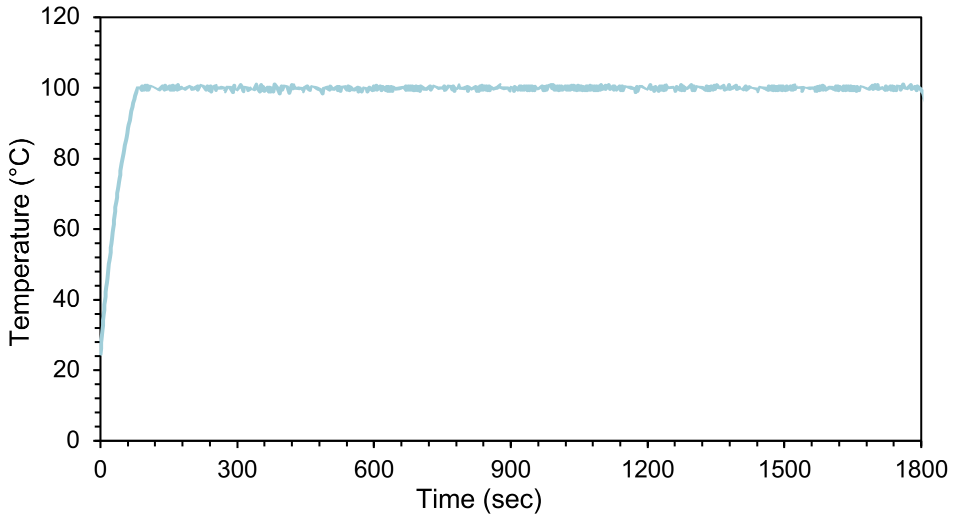

Supplementary figure 55 | Temperature profile of AC@UIO-66 synthesis using 850nm 100W LED

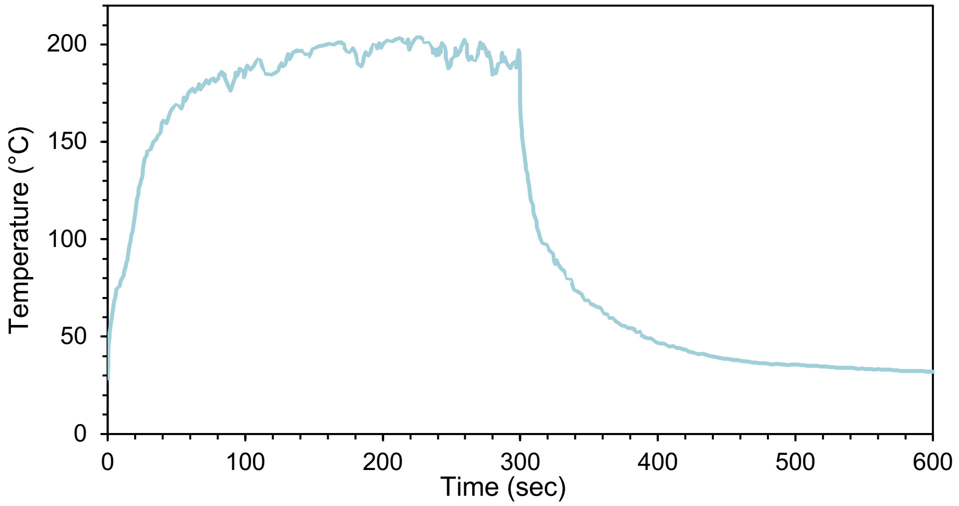

Supplementary figure 56 | Heating ability of AC@UIO-66 irradiated by 850nm 100W LED

13.3 Supplementary Figure 57-58: Photothermal synthesis using graphene oxide

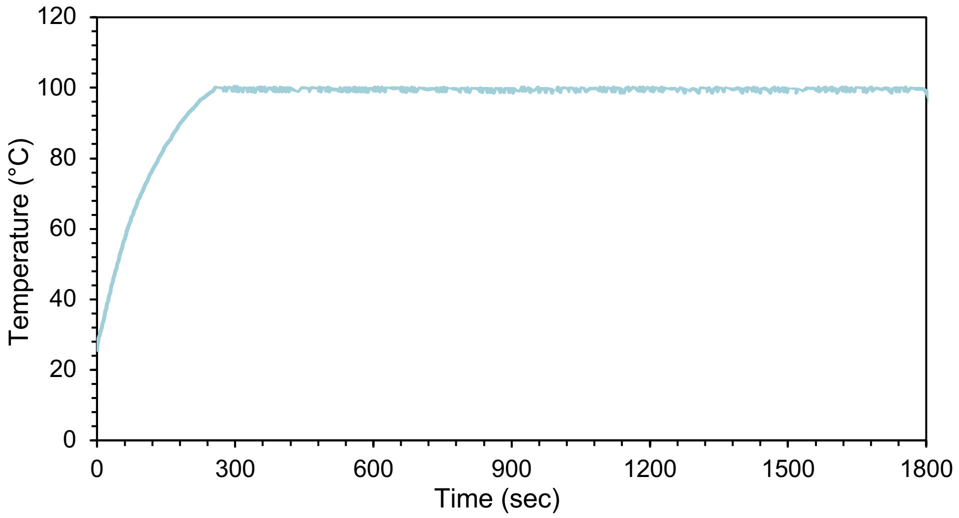

Supplementary figure 57 | Temperature profile of GO@UIO-66 synthesis using 850nm 100W LED

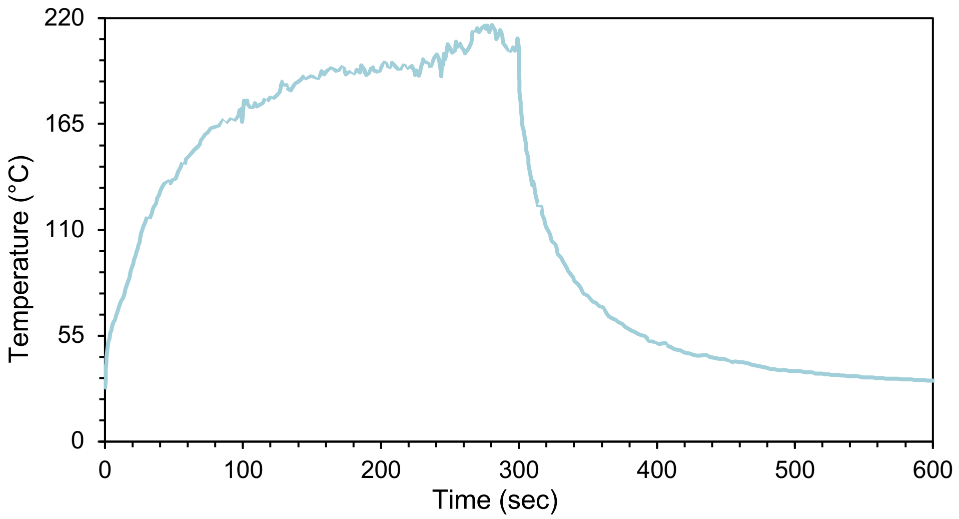

Supplementary figure 58 | Heating ability of GO@UIO-66 irradiated by 850nm 100W LED

13.4 Supplementary Figure 59-61: Photothermal synthesis using $AuBPs_{660}$

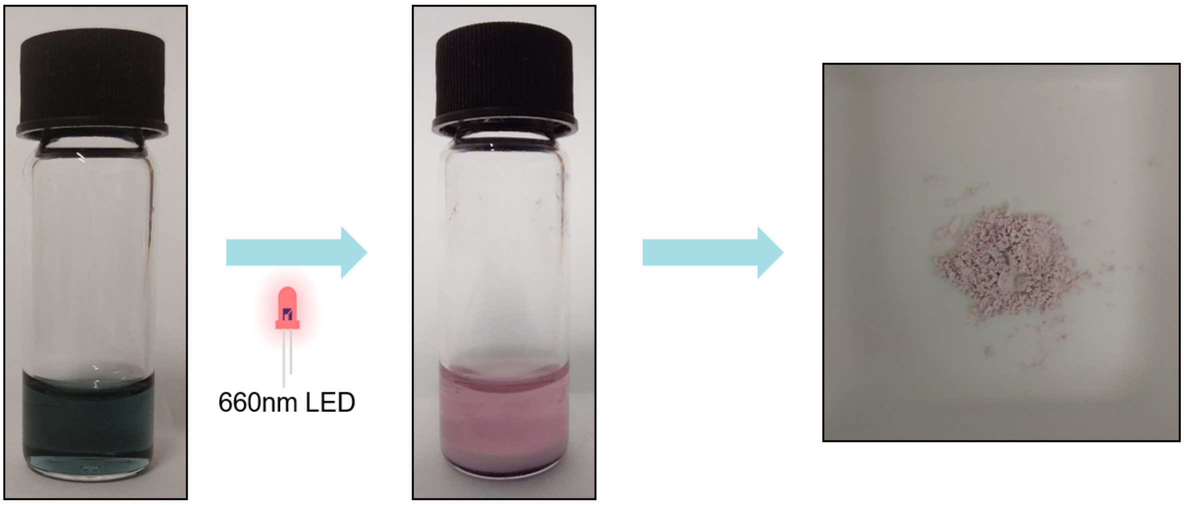

Supplementary figure 59 | Scheme of $AuBP_{660}$@UIO-66 synthesis using 660nm LED. 2 OD of $AuBP_{660}$, 1 ml, 20 minutes, 100 °C

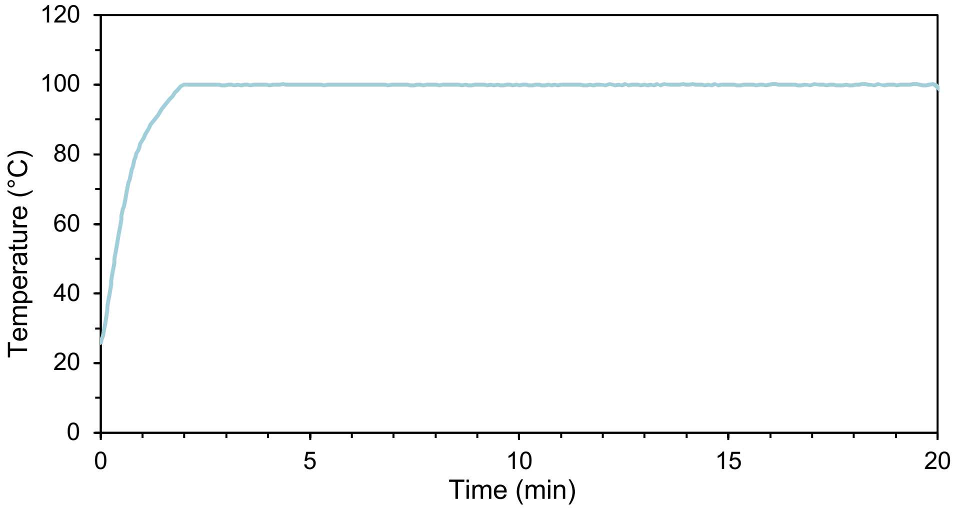

Supplementary figure 60 | Temperature profile of $AuBP_{660}$@UIO-66 photothermal synthesis using 660nm 100W LED

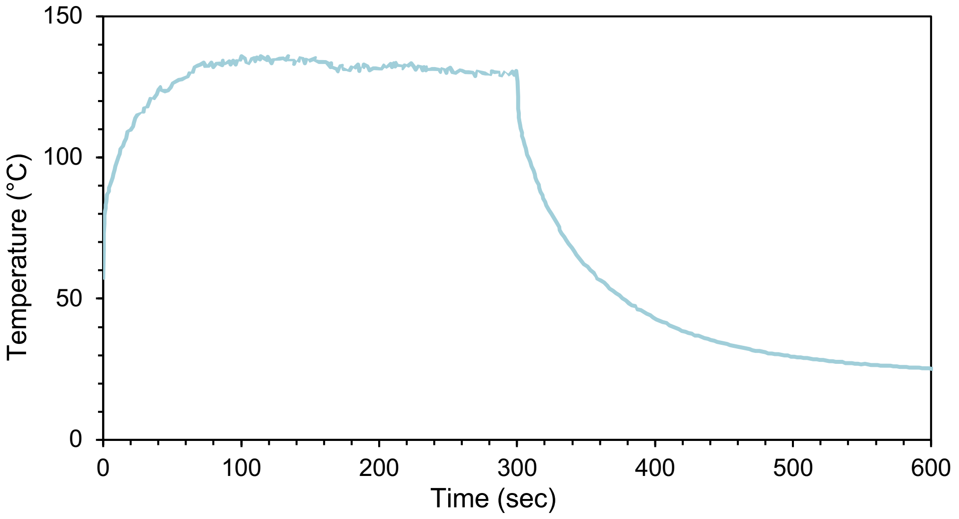

Supplementary figure 61 | Heating ability of $AuBP_{660}$@UIO-66 irradiated with 660nm 100W LED

13.5 Supplementary Figures 62-66: Photothermal synthesis using AuNS

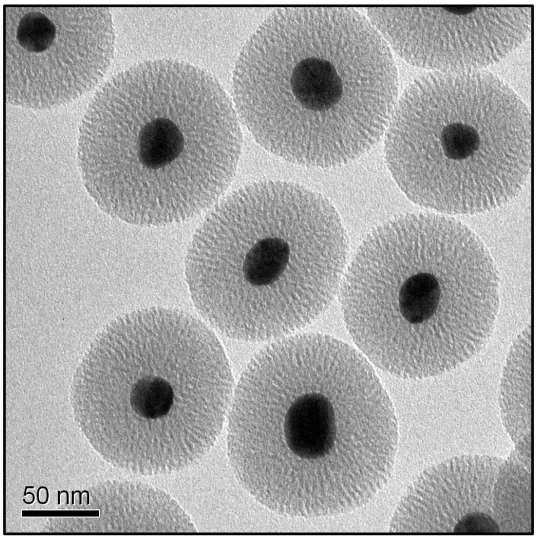

Supplementary figure 62 | TEM images of encapsulated AuNS.

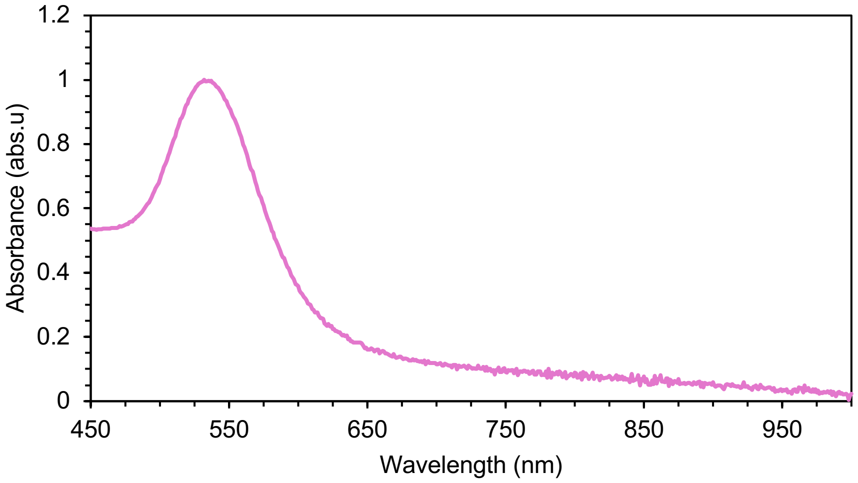

Supplementary figure 63 | UV-vis spectrum of AuNS.

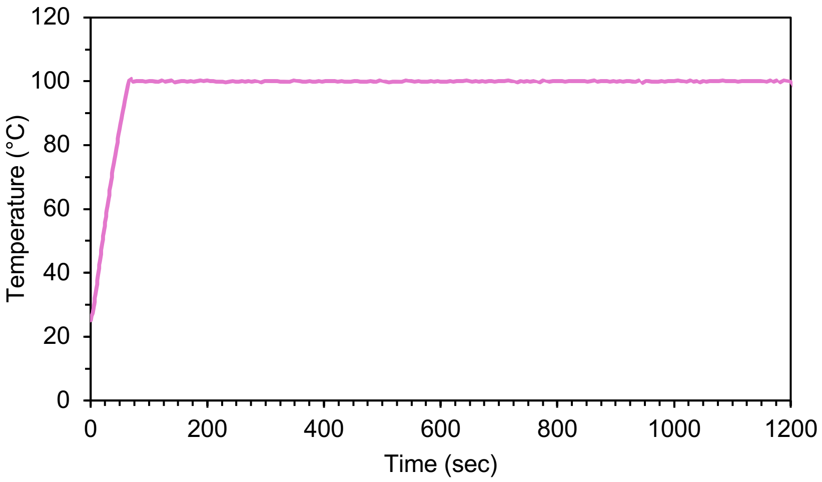

Supplementary figure 64 | Temperature profile of AuNS@UIO-66 photothermal synthesis using 520nm 100W LED.

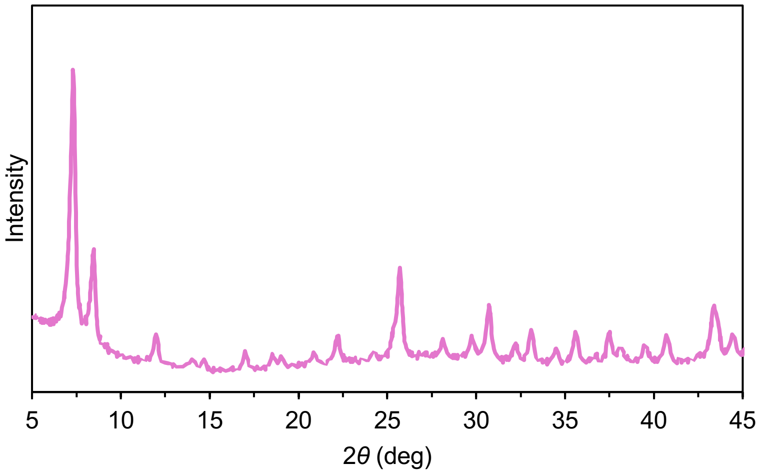

Supplementary figure 65 | PXRD pattern of AuNS@UIO-66.

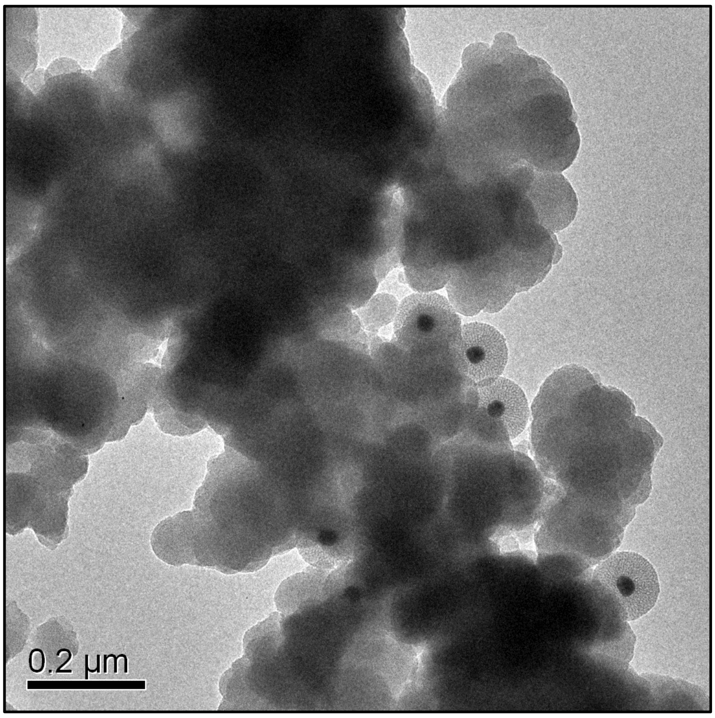

Supplementary figure 66 | TEM image of AuNS@UIO-66.

13.6 Supplementary Figures 67-71: Photothermal synthesis using AuNR

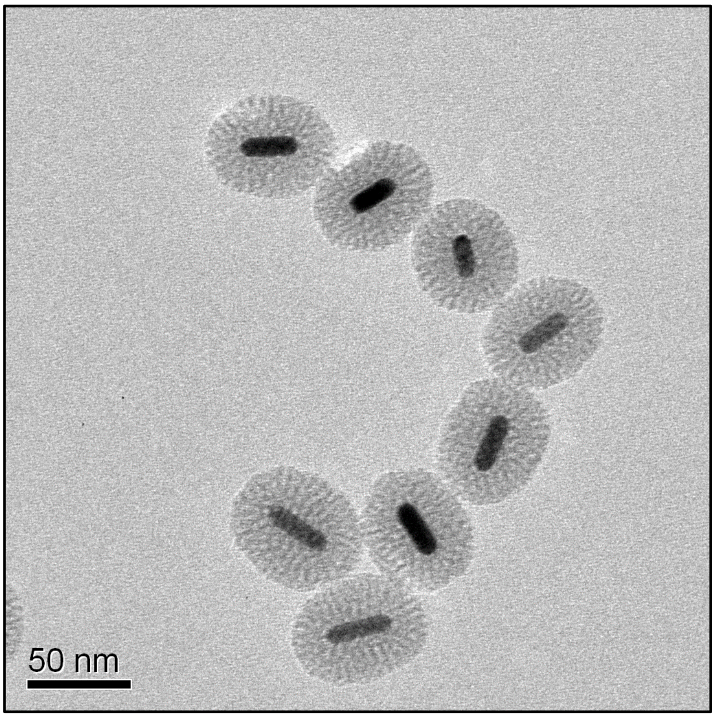

Supplementary figure 67 | TEM images of encapsulated AuNR.

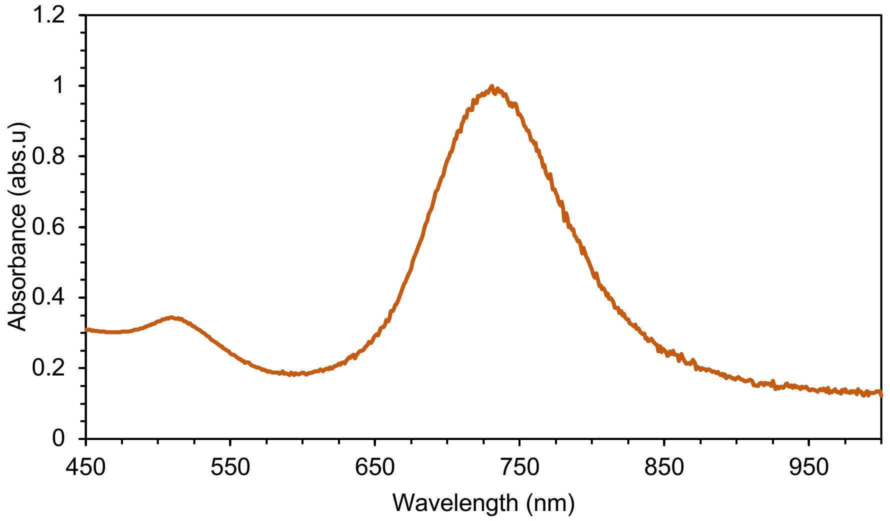

Supplementary figure 68 | UV-vis spectrum of AuNR.

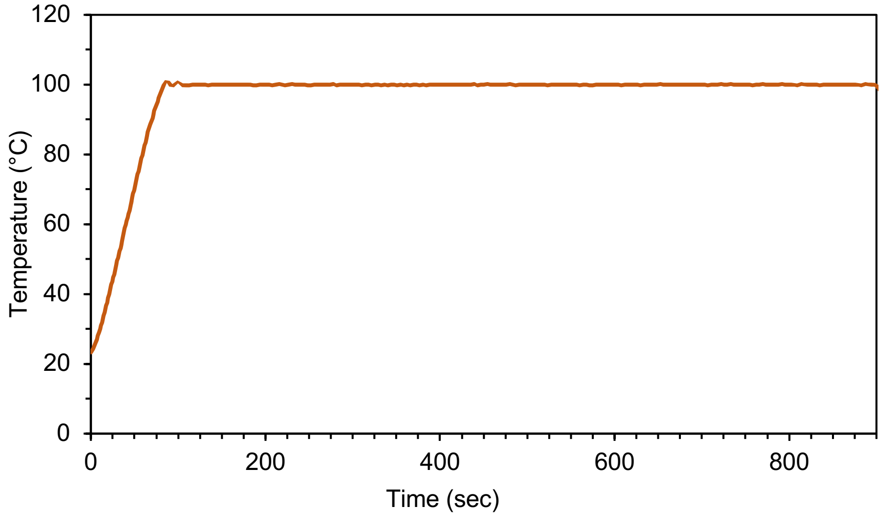

Supplementary figure 69 | Temperature profile of AuNR@UIO-66 photothermal synthesis using 660nm 100W LED.

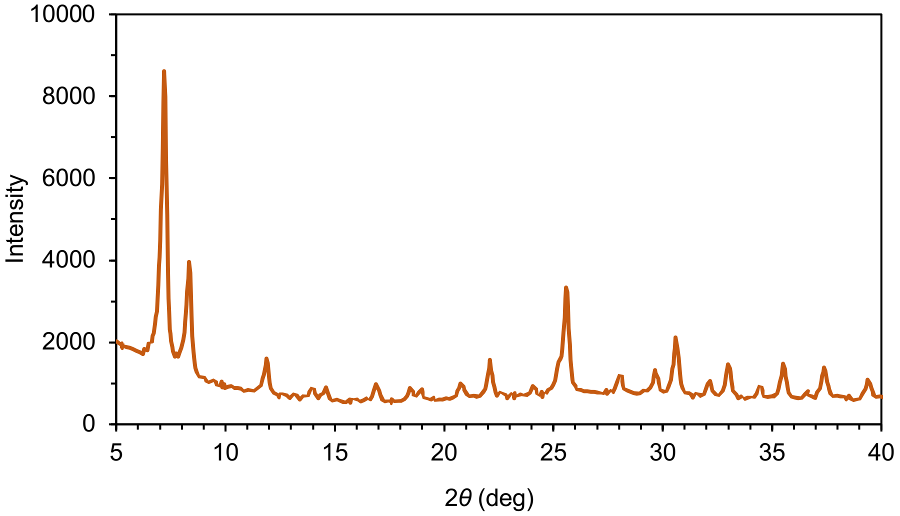

Supplementary figure 70 | PXRD pattern of AuNS@UIO-66.

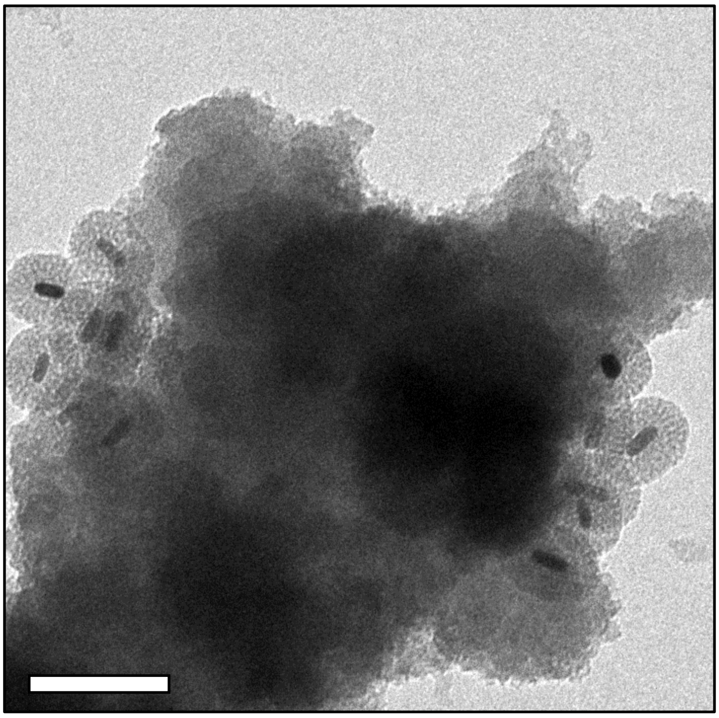

Supplementary figure 71 | TEM image of AuNR@UIO-66. White scale bar- 100 nm.

14. Supplementary Note 10: AuBP embedded UIO-66

14.1 Supplementary Figure 72: Heating ability of UIO-66 without AuBPs

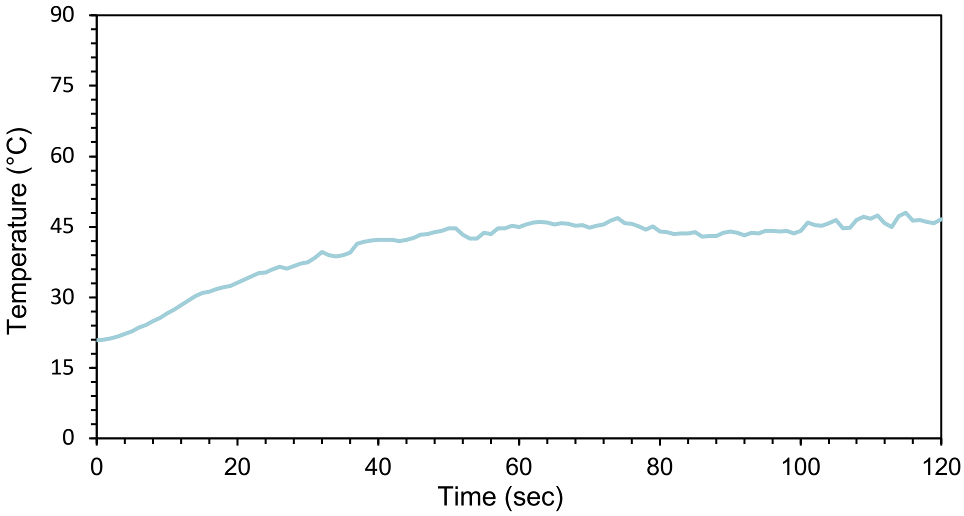

Supplementary figure 72 | UIO-66 irradiated with 850nm 100W LED for 2 minutes.

14.2 Supplementary Table 7: Heating-Cooling cycles properties

Supplementary table 7

	Temperature range	
	**40 °C-100 °C**	**40 °C-180 °C**
Duration (hours)	3	3
Number of cycles	182	87
Average time per cycle (seconds)	59.3	124.1
Average heating ramp rate	12 °C per second	6.5 °C per second

Data of heating cycles of $AuBP_{850}$@UIO-66

14.3 Supplementary Figure 73-74 Heating-Cooling cycles

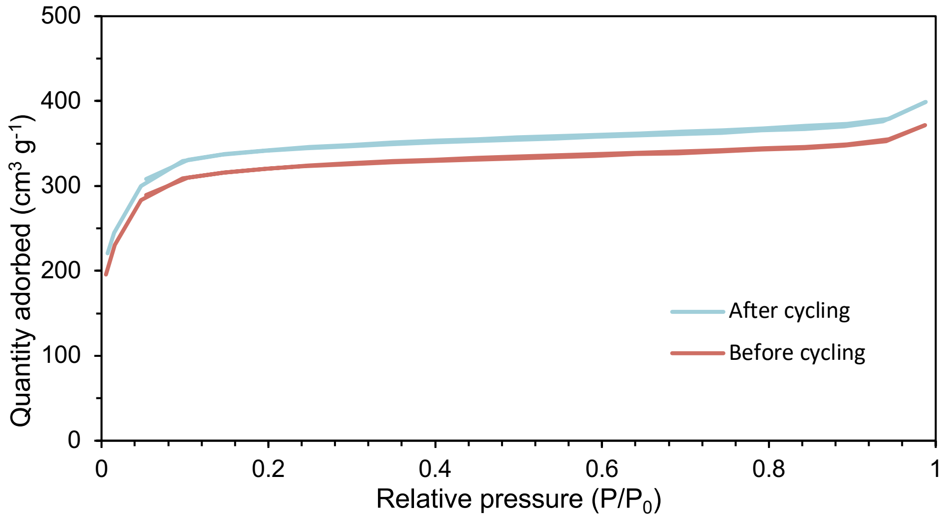

Supplementary figure 73 | N_2 isotherms of $AuBP_{850}$@UIO-66 before and after heating cycles

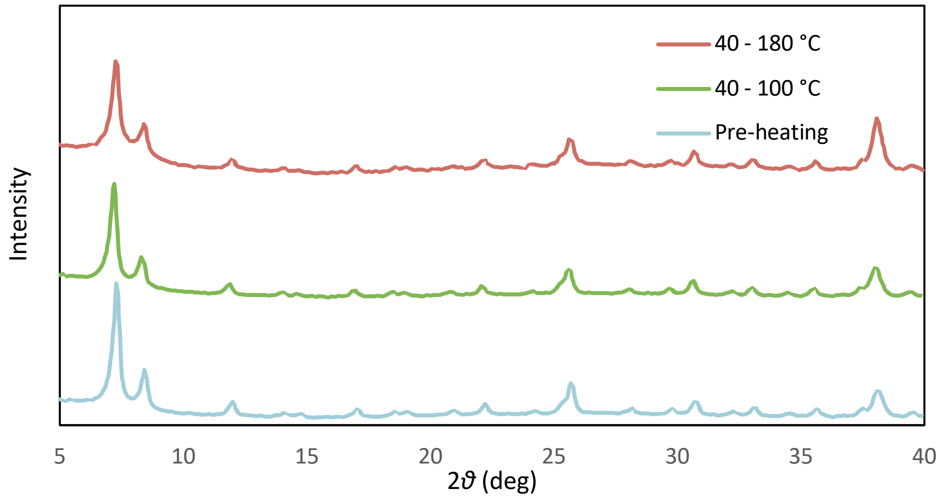

Supplementary figure 74 | PXRD patterns of $AuBP_{850}$@UIO-66 before and after heating cycles

14.4 Supplementary Figures 75-76: Water desorption

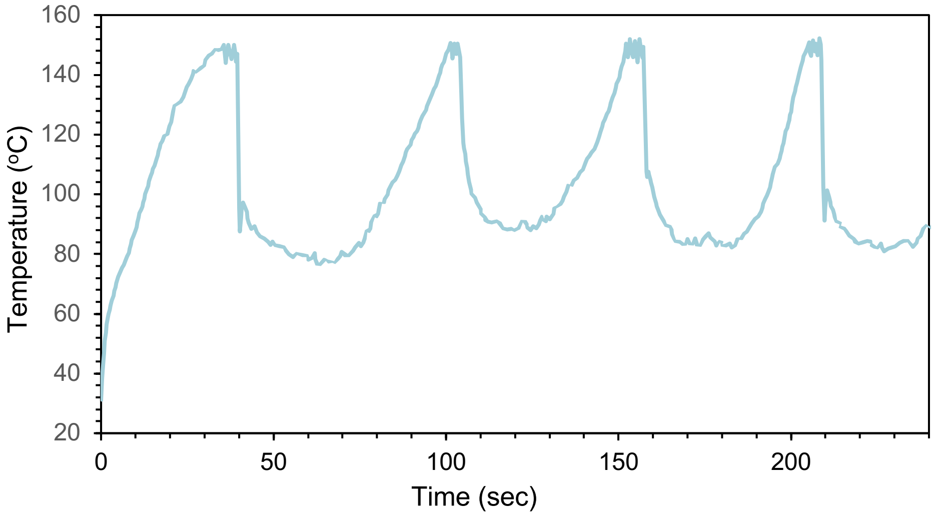

Supplementary figure 75 | Temperature profile of AuBP$_{850}$@UIO-66 with 5 OD of AuBPs, while dropping 40ul of water on it few times.

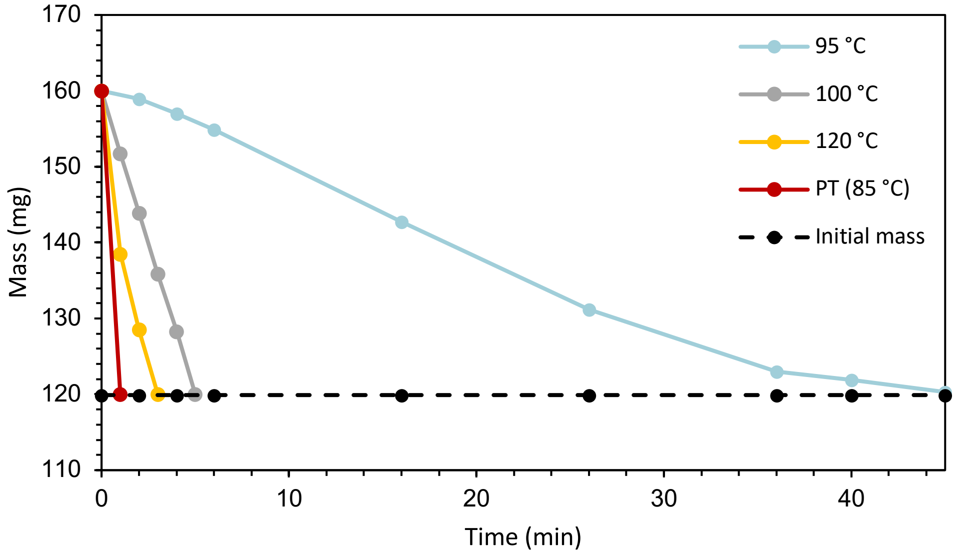

Supplementary figure 76 | The mass of UIO-66 while drying in the oven at different temperature

15. Supplementary Note 11: Photothermal activation

Procedure

Photothermal activation procedure: desired amount of dry AuBP@MOF was put inside a BET sample tube, the tube was connected to a pump. The MOF was irradiated by a 100W 850nm LED for 5 minutes, the tube with the sample was taken to BET instrument for surface area checking

15.1 Supplementary Figure 64: Photothermal activation set-up

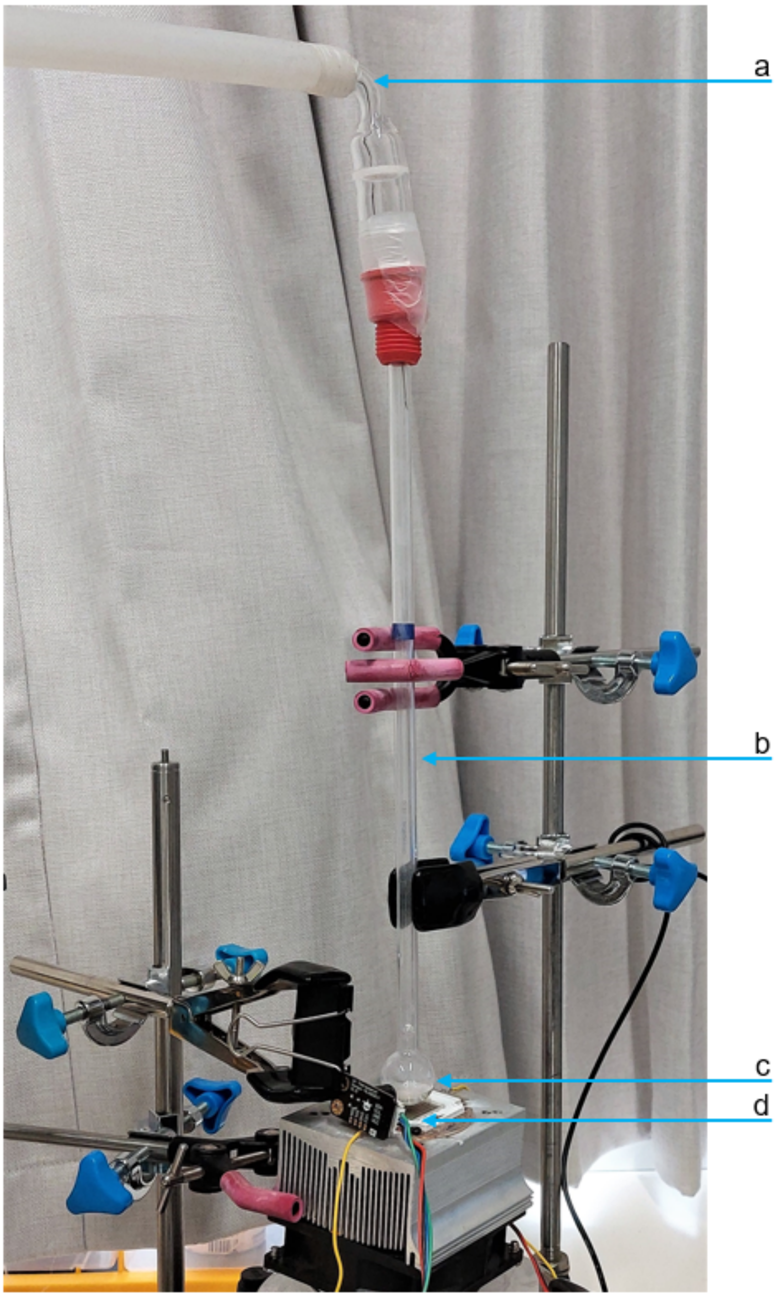

Supplementary figure 77 | Photothermal MOF activation set-up. a. Pump's pipe. b. BET tube. c. dry $AuBP_{850}$@UIO-66. d. 100W 850nm LED.

16. Supplementary Note 12: UIO-66@UIO-66

16.1 Supplementary Figure 78: Temperature profile UIO-66@UIO-66 synthesis cycles.

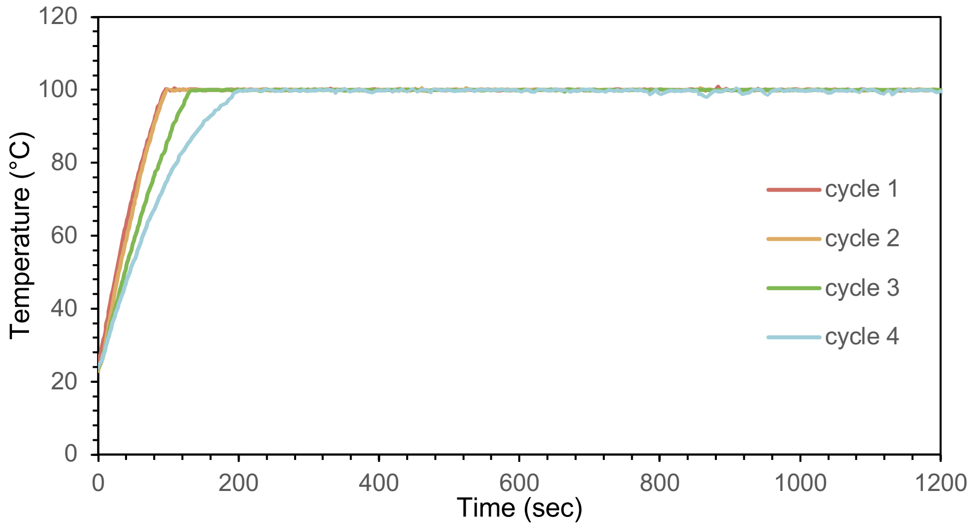

Supplementary figure 78 | Temperature profiles of UIO-66@UIO-66 synthesis cycles

16.2 Supplementary Table 8: UIO-66@UIO-66 synthesized masses

Supplementary table 8

Cycle	Total mass (mg)	Δ mass (mg)
1	6.9	-
2	12.7	5.8
3	18.3	5.6
4	24.1	5.8

masses of the product of UIO-66@UIO-66 synthesis cycles

17. Supplementary references

1. Katz, M. J. *et al.* A facile synthesis of UiO-66, UiO-67 and their derivatives. *Chem. Commun.* **49**, 9449–9451 (2013).

2. Cavka, J. H. *et al.* A new zirconium inorganic building brick forming metal organic frameworks with exceptional stability. *J. Am. Chem. Soc.* **130**, 13850–13851 (2008).

3. Kraš, A. & Milošev, I. The Aqueous Chemistry of Zirconium as a Basis for Better Understanding the Formation of Zirconium Conversion Coatings: Updated Thermodynamic Data. *J. Electrochem. Soc.* **170**, 021508 (2023).

4. Singhal, A., Toth, L. M., Lin, J. S. & Affholter, K. Zirconium(IV) tetramer/octamer hydrolysis equilibrium in aqueous hydrochloric acid solution. *J. Am. Chem. Soc.* **118**, 11529–11534 (1996).

5. Zhukov, A. V., Chizhevskaya, S. V., Phyo, P. & Panov, V. A. Heterophase Synthesis of Zirconium Hydroxide from Zirconium Oxychloride. *Inorg. Mater.* **55**, 994–1000 (2019).

6. Chen, J. *et al.* Sensing of Hydrogen Sulfide Gas in the Raman-Silent Region Based on Gold Nano-Bipyramids (Au NBPs) Encapsulated by Zeolitic Imidazolate Framework-8. *ACS Sensors* **5**, 3964–3970 (2020).
