# Supplementary material for: Light-induced MOF synthesis enabling composite photothermal materials
Source: Nat Commun. 2024 Feb 7;15:1154. doi: 10.1038/s41467-024-45333-9 (PMC10850081; doi:10.1038/s41467-024-45333-9)
Supplement: Supplementary file 4 — Description of Additional Supplementary Files [file 41467_2024_45333_MOESM4_ESM.pdf]

Description of Additional Supplementary Files

Supplementary Movie Legends

Supplementary Movie 1: Side-by-side videos depict the photothermal synthesis of UIO66. The total volume used was 2 ml with an AuBPs concentration of 5 OD. The 100W 850nm LED was irradiated for 9 minutes. The video was accelerated x30. The thermal video, filmed with a FLIR ONE PRO camera, appears on the right, while the standard recording is on the left.

Supplementary Movie 2: Dry AuBP@UIO-66, initially with an AuBP850 concentration of 5 OD, was placed on a glass slide above a 100W 850nm LED. The LED irradiated it for 80 seconds. The video was accelerated x2. Thermal imagery (recorded with an FLIR ONE PRO camera), featuring temperature readings.
